# Advanced Strategies for Enhancing the Biocompatibility and Antibacterial Properties of Implantable Structures

**DOI:** 10.3390/ma18040822

**Published:** 2025-02-13

**Authors:** Oleg Mishchenko, Kristina Volchykhina, Denis Maksymov, Olesia Manukhina, Maksym Pogorielov, Mykola Pavlenko, Igor Iatsunskyi

**Affiliations:** 1 Department of Dentistry of Postgraduate Education, Zaporizhzhia State Medical and Pharmaceutical University, 26 Marii Prymachenko Blvd., 69035 Zaporizhzhia, Ukraine; kristinavolchihina@gmail.com (K.V.); maximovdenis0064@gmail.com (D.M.); manoln2@gmail.com (O.M.); 2 Insitute of Atomic Physics and Spectroscopy, University of Latvia, 3 Jelgavas Str., LV-1004 Riga, Latvia; m.pogorielov@gmail.com; 3 NanoBioMedical Centre, Adam Mickiewicz University, Wszechnicy Piastowskiej Str. 3, 61-614 Poznan, Poland; mykpav@amu.edu.pl

**Keywords:** osseointegration, titanium implants, peri-implantitis, surface modification, bacterial contamination, biocompatibility, plasma electrolytic oxidation (PEO), ion implantation, antibacterial surfaces

## Abstract

This review explores the latest advancements in enhancing the biocompatibility and antibacterial properties of implantable structures, with a focus on titanium (Ti) and its alloys. Titanium implants, widely used in dental and orthopedic applications, demonstrate excellent mechanical strength and biocompatibility, yet face challenges such as peri-implantitis, a bacterial infection that can lead to implant failure. To address these issues, both passive and active surface modification strategies have been developed. Passive modifications, such as altering surface texture and chemistry, aim to prevent bacterial adhesion, while active approaches incorporate antimicrobial agents for sustained infection control. Nanotechnology has emerged as a transformative tool, enabling the creation of nanoscale materials and coatings like TiO_2_ and ZnO that promote osseointegration and inhibit biofilm formation. Techniques such as plasma spraying, ion implantation, and plasma electrolytic oxidation (PEO) show promising results in improving implant integration and durability. Despite significant progress, further research is needed to refine these technologies, optimize surface properties, and address the clinical challenges associated with implant longevity and safety. This review highlights the intersection of surface engineering, nanotechnology, and biomedical innovation, paving the way for the next generation of implantable devices.

## 1. Introduction

The groundbreaking discovery of osseointegration by Brånemark and colleagues in 1969 catalyzed significant advancements in the field of implantation [1]. Titanium (Ti) implants have demonstrated excellent performance in clinical settings, owing to their robust mechanical properties and superior biocompatibility. However, challenges persist in achieving optimal osseointegration, particularly for patients with compromised health conditions [2,3]. Dental implants play a crucial role in effectively managing various orthopedic disorders, but complications such as implant-associated infections remain frequent and challenging to address [4]. In dentistry, peri-implantitis refers to an inflammatory condition affecting the tissues surrounding an osseointegrated implant, which can result in the degradation of supporting bone and potential implant failure. Reports indicate a prevalence rate ranging between 5% and 63.4%, a variability attributed to differences in study methodologies and risk factors assessed [5]. Regardless of these variations, peri-implantitis represents a major issue in implantology, primarily due to the high infection risk during implant placement.

Key bacterial species implicated in peri-implant inflammation include *Staphylococcus aureus* and *Staphylococcus epidermidis*. Additionally, a diverse range of microorganisms, such as *Prevotella intermedia*, *Porphyromonas gingivalis*, *Aggregatibacter actinomycetemcomitans*, *Bacteroides forsythus*, *Treponema denticola*, *Prevotella nigrescens*, *Peptostreptococcus micros*, and *Fusobacterium nucleatum*, have been associated with the condition [6,7]. The process of bacterial contamination begins with the attachment of microbial cells to the implant’s surface, followed by proliferation and the development of an extracellular biofilm matrix. Once mature, bacteria within the biofilm can disperse and colonize new areas, further exacerbating infection risks [8]. Consequently, designing implant surfaces that inhibit bacterial adhesion and biofilm formation is critical for ensuring implant success.

Historically, research has focused on the interaction between implant surfaces and bone tissue. More recently, it has become evident that altering the surface texture or chemical composition of implants can confer antibacterial properties. With the rise of antibiotic-resistant bacterial strains, early-stage interactions between implant materials and microbial cells have gained heightened attention [9].

Current approaches to preventing peri-implantitis aim to either eliminate bacteria (bactericidal action) or impede their adhesion and biofilm development (anti-adhesive action). These strategies can be broadly categorized into passive and active surface modifications. Passive modifications involve structural or chemical alterations designed to deter bacterial adherence without releasing antimicrobial agents. Conversely, active modifications rely on the release of pharmacologically active antibacterial substances [10,11].

Recent advancements in nanotechnology have explored innovative methods to simultaneously minimize bacterial contamination and enhance osseointegration. For instance, Tsimbouri et al. [12] developed a TiO_2_ nanowire-based material that improves bone attachment while reducing bacterial colonization. Similarly, Colon et al. [13] synthesized sintered TiO_2_ and ZnO nanospheres to achieve comparable outcomes. Cheng et al. [14] investigated titanium oxide nanotubes doped with silver and strontium via hydrothermal treatment, whereas Huo et al. [15] employed anodization and hydrothermal techniques to create Zn-doped TiO_2_ nanotubes. Ferraris et al. [16] further demonstrated that chemically treating titanium surfaces to incorporate micro- and nanostructures functionalized with silver nanoparticles could enhance osseointegration while imparting antimicrobial properties.

In contemporary medical practice, addressing issues related to peri-implantitis remains a priority. This inflammatory condition, driven by bacterial infections, leads to bone loss and may ultimately result in implant failure. Research emphasizes the importance of developing advanced surface coatings to prevent bacterial adhesion and biofilm formation. The incorporation of nanoscale materials, such as nanotubes and nanoparticles, has shown promise in improving osseointegration and offering antibacterial benefits. These advancements underscore the critical need for innovative implant surface modifications to reduce complications and enhance the safety and longevity of dental implants in clinical applications.

## 2. Osseointegration and Surface Quality Management

The success of osseointegration hinges on two fundamental processes: mechanical fixation of the implant within the bone and the biological interaction of cells with the implant surface, which culminates in the formation of mature bone tissue [17]. These processes are vital for enabling the implant to bear and redistribute functional loads, thereby restoring the patient’s masticatory function. Numerous strategies have been developed to modify implant surfaces to create conditions conducive to bone tissue development. Traditional methods, such as calcium phosphate coatings, are valued for their osteoconductive properties, and the incorporation of antibacterial agents, including silver, has been shown to reduce bacterial complications [18,19]. For instance, Besinis et al. [19] utilized plasma sputtering to deposit a HAR coating doped with Ag_2_O and SrO on titanium, finding that silver effectively inhibited *Pseudomonas aeruginosa*. However, its release adversely affected osteoblast activity, an issue mitigated by adding SrO to the coating.

Nanoscale surface modifications have gained prominence for their ability to alter cell behavior. The influence of surface properties on cellular activity has been recognized for over a century [20], with the term “contact integration” emerging in the 1950s and 1960s. Modern techniques, such as photolithography, colloid lithography, and polymer phase separation, have elucidated how surface features at the micro- and nanoscale impact cellular responses [21,22,23,24]. For example, zirconium nanoparticles used to modify implant surfaces have shown improved fibrinogen absorption and osteogenic cell interactions compared to conventional machining [25]. Patelli et al. [26] demonstrated that 220 nm silicon granules incorporated in PLGA coatings increased osteoblast adhesion by 20% compared to commercial implants. Similarly, nano-TiO_2_ coatings incorporating hydroxyapatite stimulated bone tissue growth in vivo, enhancing implant fixation [27].

In addition to promoting osteogenesis, nanosurfaces can stimulate epithelial and connective tissue growth. Xu et al. [28] applied plasma electrooxidation and selective laser melting to create nanoscale patterns on calcium phosphate coatings, which increased epithelial cell proliferation and gene expression. A search of PubMed revealed over 10,000 publications in the past 15 years addressing calcium phosphate coatings’ roles in osseointegration, osteogenic cell activity, and MSC differentiation. However, the effects of surface roughness and chemical composition remain complex, with some studies showing a positive correlation between micrometer-scale modifications and osseointegration, while others report no clear relationship [29,30].

Various surface modification methods have been employed to improve osseointegration, including plasma sputtering, chemical vapor deposition (CVD), physical vapor deposition (PVD), and ion implantation [31,32,33]. PVD, for instance, involves the deposition of thin, dense coatings with strong adhesion and has been used to apply TiC and TiN coatings to titanium surfaces [34,35,36]. Plasma-based treatments, such as glow discharge, have been shown to enhance surface energy, clean biomaterial surfaces, and improve wear and corrosion resistance [37,38,39,40,41,42,43,44,45,46,47,48]. Glow discharge nitriding and carbonitriding have also been used to create diffusion-based surface layers with enhanced hardness, wear resistance, and biocompatibility [47,49].

Ion beam implantation has emerged as a powerful tool for modifying implant surfaces. This method introduces energetic ions into a substrate’s surface, improving corrosion resistance and biocompatibility. Calcium and phosphorus ion implantation, for example, enhances the biological activity of titanium, promoting calcium phosphate deposition and osteogenesis [50,51,52,53,54,55,56,57,58,59,60,61,62,63]. Other ions, including sodium, fluorine, and nitrogen, have been used to impart antibacterial properties, improve mechanical performance, and stimulate bone tissue regeneration [64,65,66,67,68,69,70,71,72,73,74,75].

Nanoscale materials, such as TiO_2_ and ZrO_2_ nanotubes, have been shown to influence cell behavior through mechanotransduction, converting mechanical stimuli into biochemical signals that regulate gene expression [76,77,78,79,80,81,82]. The diameter of these nanotubes plays a critical role in MSC differentiation and osteoblast proliferation, with diameters of 70–100 nm typically promoting optimal outcomes [83,84,85,86,87,88,89,90,91,92,93,94,95,96,97,98,99,100,101,102,103,104,105,106,107,108,109]. However, conflicting findings suggest that further research is needed to establish definitive design parameters [110,111,112,113,114,115,116,117,118,119,120,121].

Preventing bacterial adhesion and biofilm formation is a crucial challenge in implantology. Strategies include bactericidal coatings releasing silver ions or antibiotics and physical modifications that deter microbial attachment [3,122,123,124,125,126,127,128,129,130]. For instance, Ercan et al. demonstrated that nanoscale modifications to titanium surfaces significantly reduce bacterial adhesion [131,132,133,134,135,136,137,138,139,140,141,142]. Laser-induced periodic surface structures (LIPSSs) have also been explored to create multifunctional surfaces that support cellular attachment while inhibiting bacterial growth [143,144,145,146,147,148,149,150,151,152,153,154,155,156,157,158,159,160,161,162].

Despite the widespread adoption of calcium phosphate coatings and other surface modifications, many questions remain about nanoscale features’ optimal size and composition for long-term osseointegration. While studies have shown that nanosized hydroxyapatite improves protein adsorption and osteoblast adhesion, the interplay between chemical composition, surface roughness, and mechanical properties warrants further investigation [163,164,165,166,167,168,169,170,171,172,173,174,175]. For instance, Mendes et al. [172] demonstrated that nanocrystalline modifications accelerate osseointegration, while Schliephake et al. [174] highlighted the importance of nanoscale roughness in promoting bone formation. Additionally, the effects of nanosurface features on macrophages and other immune cells remain poorly understood, underscoring the need for more comprehensive studies [112,113,114,115,116,117,118]. While significant progress has been made in implant surface engineering, further research is essential to optimize designs that enhance osseointegration, reduce infection risk, and ensure long-term implant success.

### Reconciling Conflicting Findings on Surface Roughness and Coating Compositions

Surface roughness and coating composition are critical factors influencing implantable structures’ biocompatibility, osseointegration, and antibacterial properties. However, contradictory findings in the literature regarding the optimal parameters for these features present challenges for developing standardized implant surfaces. This section summarizes the discrepancies, examines potential reasons for these inconsistencies, and provides insights into how these variations can be interpreted and addressed.

Some studies indicate moderate roughness (1–2 μm) promotes optimal osseointegration by increasing surface area and enhancing bone–implant interactions. For instance, roughness of approximately 1.5 μm significantly improved osteoblast attachment and proliferation. Conversely, other research suggests excessive roughness in the cervical region (>2 μm) increases the risk of bacterial colonization and peri-implantitis. For example, Ferraris et al. reported that highly roughened surfaces provided favorable conditions for biofilm formation, potentially compromising long-term implant stability.

Similarly, calcium-phosphate-based coatings, such as hydroxyapatite (HA), have demonstrated strong osteoconductive properties and enhanced bone regeneration. However, some studies highlight issues with coating delamination and reduced mechanical stability under prolonged in vivo conditions. Antibacterial coatings incorporating silver or zinc ions effectively reduce bacterial adhesion but occasionally impair osteoblast activity. It was noted that silver-doped coatings reduced bacterial contamination but inhibited cell proliferation at higher ion concentrations.

Several factors contribute to the conflicting findings in the literature. Variability in experimental conditions, such as differences in the base materials used (e.g., titanium vs. titanium alloys), affects the outcomes of surface modifications. Different methodologies, such as plasma spraying, ion implantation, or anodization, result in varying surface chemistries and morphologies. Variations in methods for assessing surface roughness (e.g., profilometry vs. atomic force microscopy) and coating properties (e.g., XRD vs. SEM) may yield inconsistent results. Clinical and biological factors influence implant performance, including patient health status and location. For example, systemic conditions like diabetes, osteoporosis, and mechanical stresses in load-bearing regions can lead to divergent outcomes. Additionally, the short follow-up periods in many studies fail to capture long-term performance, and small sample sizes and limited in vivo studies reduce the generalizability of findings.

Understanding these inconsistencies is crucial for advancing implant design and ensuring clinical success. Developing uniform guidelines for experimental procedures and measurement techniques is essential for guaranteeing comparability across studies. Combining antibacterial and osteoconductive strategies in a single coating may mitigate trade-offs between biofilm resistance and osseointegration. Conducting extended in vivo studies with large sample sizes will provide more robust data on the durability and safety of modified implant surfaces. Collaboration between materials scientists, microbiologists, and clinicians is necessary to design surfaces that address mechanical and biological requirements. By systematically summarizing and critically evaluating the conflicting findings, this review underscores the importance of nuanced interpretations and the need for ongoing research to optimize surface modifications for implantable structures.

## 3. PEO, Aspects of Morphology

Surface roughness and coatings on dental implants have been extensively studied in vitro, yet the number of clinical publications providing detailed insights into implant surfaces remains limited. Furthermore, much of the available data often reflect the combined effects of multiple factors influencing dental implantation success rather than findings from randomized, controlled clinical and experimental studies [175]. Consequently, translating in vitro findings into clinically relevant conclusions remains challenging.

Research indicates that an average surface roughness of at least 1 micron improves bone maintenance and implant survival. Systematic reviews over the past decade support this conclusion [176,177]. Studies assessing histomorphometric parameters have consistently shown that implants with rough surfaces demonstrate superior osseointegration compared to machined titanium implants [177]. Increased surface roughness enhances the bone–implant connection by enlarging the surface area available for interaction at the micron level.

However, excessive roughness, particularly in the cervical region of the implant, has been linked to a higher risk of peri-implantitis [178]. Thus, moderate roughness, typically ranging from 1 to 2 microns, is widely considered optimal for dental implants [179].

Manufacturers have developed numerous techniques to create rough surfaces on dental implants [180]. Among the most common are sandblasting (1) and acid etching (2). (1) This technique involves propelling hard ceramic particles at high velocities onto the implant surface, creating irregularities in the form of bumps and tears. However, this process may result in sharp-angled defects and residual foreign particles embedded in the surface. (2) To mitigate the issues associated with sandblasting, implants are often subjected to additional acid treatment using strong acids such as HF, HCl, or HNO_3_. This step generates a characteristic rough surface, free from foreign particle contamination, and is known to promote osseointegration.

To address the limitations of sandblasting alone, implants are typically subjected to an additional acid treatment using strong acids such as HF, HCl, or HNO_3_. This combined process results in a distinctive rough surface that enhances osseointegration by promoting better bone–implant interactions [181]. Despite the extensive research on surface modifications, clinical studies specifically examining surface coatings are relatively sparse. A meta-analysis involving 19 large animal models demonstrated that coatings significantly improved the quality of bone–implant contact compared to uncoated implants. Among these, inorganic coatings yielded a 14.7% improvement in integration, extracellular matrix coatings showed a 10.0% enhancement, and peptide coatings provided a 7.1% improvement [181]. However, a separate meta-analysis of clinical studies suggested that hydroxyapatite coatings do not significantly affect implant survival rates.

Several strategies have been proposed in the scientific literature, patents, and clinical studies to improve the bonding ability of titanium surfaces with bone tissue. These include modifying surface topography, applying bioactive coatings, and using chemical or electrochemical treatments to create biologically active oxide layers [182]. These strategies are based on the well-established understanding that surface roughness, chemical composition, and surface charge are critical factors influencing the biological interactions of implant surfaces with surrounding tissues.

Recent research has also focused on biological functionalization, which involves attaching specific biological molecules to implant surfaces to enhance their interaction with bone tissue [183]. Additionally, the incorporation of nanofeatures has been explored to further improve cell-stimulating capabilities, reduce bacterial colonization, and impart antibacterial properties. For example, silver nanoparticles have been used for their antimicrobial effects [184]. These advancements highlight the growing trend of integrating macro-, micro-, and nanoscale modifications to optimize implant performance and ensure long-term success in clinical applications.

Adapting the texture of an implant surface has been shown to effectively modulate cellular and tissue responses [185]. Surfaces with complex topographies, featuring simultaneous micro-, sub-micro-, and nano-roughness, enhance osseointegration. Micro- and sub-micron roughness, with dimensions comparable to the size of resorption lacunae and cells, promote osteoblast differentiation, formation of focal adhesion points, and local growth factor synthesis, thereby improving implant osseointegration. Nanoscale roughness, which aligns with the size of protein receptors and cell membranes, can further influence cell adhesion, proliferation, and spreading. However, studies indicate that nanoroughness alone, without accompanying microroughness, may not sufficiently support osteoblast differentiation and proliferation.

These findings suggest that the optimal stimulation of bone for osseointegration is achieved through a combination of roughness dimensions [186,187,188]. The ideal range for sub-micro- and micro-roughness, approximately 0.4–2 μm, strikes a balance between effective bone fixation, high osteoblast adhesion, enhanced proliferation, and increased focal adhesion points, while minimizing adverse effects such as ion release and reduced implant fixation.

Titanium plasma spraying is one technique used to achieve such surface modifications. In this method, a plasma torch ejects titanium particles in an argon environment, creating a uniform layer upon merging. However, challenges such as particle erosion, changes in microparticle shape, and metal ion leakage have been reported [189]. Another method, anodizing, forms micro- or nano-textured rough surfaces while increasing the thickness and porosity of the passivated titanium oxide layer, which enhances osseointegration [190].

Various coating methods have also been developed to modify surface roughness and improve bone attachment [191]. Hydroxyapatite, for instance, can be deposited through plasma spraying; however, such coatings are prone to delamination, leading to potential implant damage in medium-term applications [192]. Similar issues have been noted with calcium orthophosphate salt coatings. Biomimetic calcium phosphate coatings, created through immersion in synthetic body fluids using the gel-sol method, offer an alternative approach [193]. Regardless of the specific method used to induce surface roughness, these modifications promote fibronectin deposition, cellular attachment, and spreading, as evidenced by both in vitro and in vivo studies [194].

Plasma electrolytic oxidation (PEO) is an advanced surface treatment technique derived from conventional anodizing, designed to form ceramic coatings on magnesium, aluminum, and titanium alloys. These coatings provide several advantages, including enhanced wear and corrosion resistance; improved biocompatibility, biodegradability, and thermal stability; and dielectric properties [195,196].

The process typically involves treating metals or alloys in silicate, phosphate, fluoride, or aluminate-containing electrolytes. This results in coatings comprising amorphous and/or crystalline phases derived from both the substrate material and the electrolyte compounds. The formation of PEO coatings is highly complex, involving electrochemical, thermal, and plasma-chemical reactions [197]. Despite its advantages, the method faces limitations, such as high porosity, a restricted range of chemistries, and significant energy consumption. Efforts to overcome these challenges have focused on optimizing electrical parameters, including applied voltage, mode, frequency, and duty cycle [198,199,200]. Additionally, altering the composition of the electrolyte has been explored to improve the microstructure and properties of the coatings [201,202,203].

**Figure 1:**
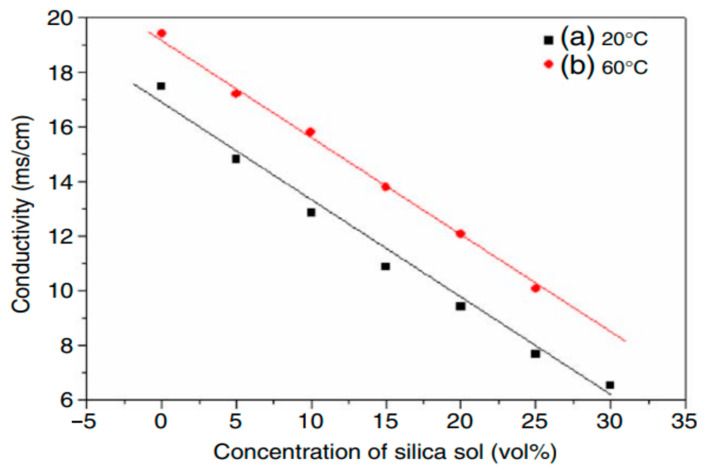
The conductivity of a 1.0 M Na_2_SiO_3_ electrolyte with varied concentration of silica sol at (**a**) 20 °C; (**b**) 60 °C. Reproduced with permission [207].

The addition of new components to the electrolyte, particularly the size of particles, can influence the PEO process. For instance, nanometer-sized particles are known to increase stress during PEO treatment compared to their micro-sized counterparts [204]. Zirconium particles, however, have demonstrated minimal impact on the coating growth rate and stress response [205]. Conversely, the use of alcohol sol (alkosol) as an additive in the electrolyte has shown a significant effect on the electrical response of the PEO process [206]. This impact is likely due to the primary solvent ethanol, which reduces the electrolyte’s conductivity and alters its electrical behavior (Figure 1) [207,208].

Recent studies indicate that increasing sol concentrations in the electrolyte significantly enhances the breakdown potential, voltage, and growth rate of coatings in the PEO process [208,209]. However, the addition of certain sols, such as aluminum oxide, has been shown to retard coating growth, resulting in lower breakdown and final voltage values [210]. This demonstrates that sols influence the PEO process more profoundly than powders, primarily due to the role of organic additives in altering the electrolyte’s composition, conductivity, and viscosity. Other factors, such as the base electrolyte, substrate material, particle properties (size and type), and electrical parameters, also contribute to the process outcome [211].

**Figure 2:**
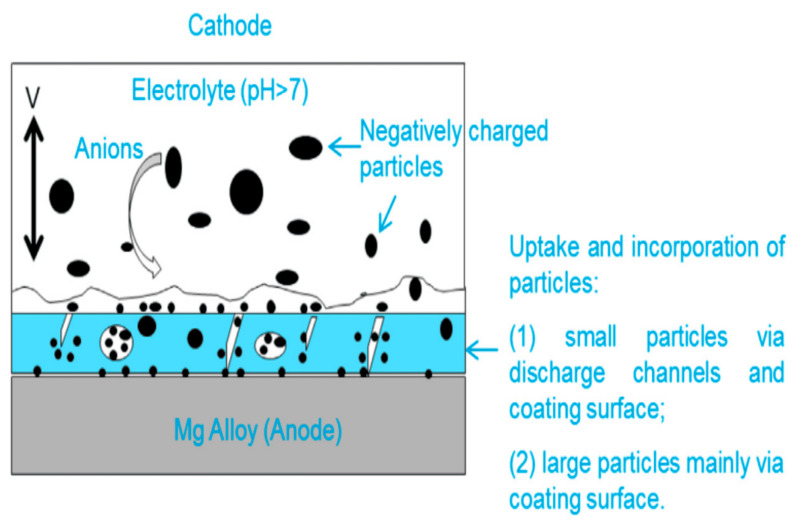
Schematic diagram of the uptake and incorporation mechanism of particles into PEO coating. Reproduced with permission [212].

Particle incorporation from the electrolyte into the coating occurs via two proposed mechanisms: absorption and inclusion (Figure 2). Absorption involves negatively charged particles being driven into the coating by the breakdown potential, leading to precipitation/adsorption in areas of enhanced anodic dissolution and reprecipitation of conversion products [211,212]. Particle size is critical, as nanosized hydroxyapatite (HA) particles penetrate deeper into the coating compared to larger microsized particles, which are unable to pass through surface pores effectively [213].

Particles incorporated into the coating during the PEO process can be either reactive or inert, depending on various factors such as the substrate, electrical parameters, electrolyte composition, and particle properties (e.g., size, melting point, and chemical stability). Adjustments to the electrical parameters can influence the mode of particle incorporation [214]. For example, inert and reactive incorporation of ZrO_2_ particles has been observed under identical electrical conditions across different electrolytes [215].

Smaller particles with lower melting points are more readily intercalated into the coating [216,217]. However, even particles with high chemical stability and low melting points can achieve inert inclusion under specific conditions. In some cases, hard sintering may occur, resulting in the coalescence of particle boundaries with the surrounding oxide matrix [218,219].

## 4. Effect of Adding Particles on Coating Composition, Microstructure, and Morphology

The composition of the PEO electrolyte is a crucial factor influencing the microstructure and morphology of the oxide layer. The introduction of various particles into the electrolyte affects the phase composition, pore characteristics, thickness, and density of the coating. Typically, particles are added directly to the electrolyte as a powder or sol, with the main challenge being achieving a uniform dispersion. To address this, the zeta potential is used to evaluate the surface charge of particles and their resistance to aggregation in a specific solution [220].

**Figure 3:**
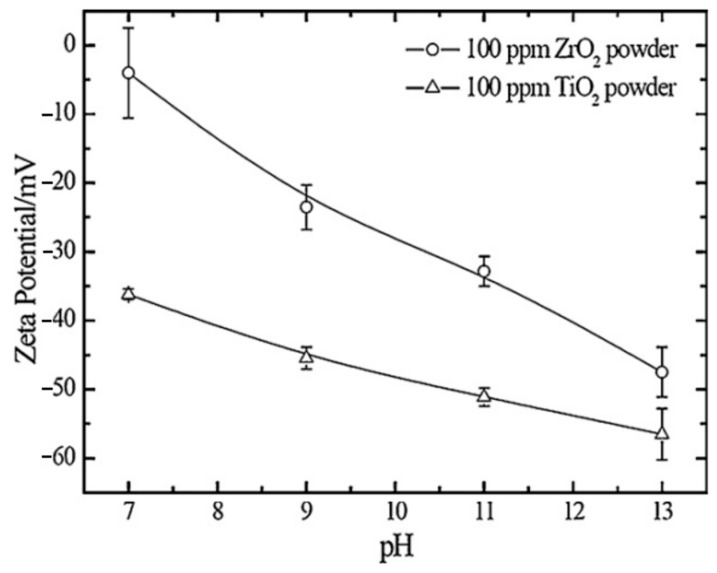
Zeta potentials of ZrO_2_ and TiO_2_ powders at different pH levels in alkaline fluoride-based electrolyte. Reproduced with permission [222].

The zeta potential magnitude indicates the degree of electrostatic repulsion between particles. A higher absolute zeta potential value corresponds to greater particle stability, inhibiting aggregation and precipitation within the electrolyte [221]. Negatively charged particles, which exhibit a negative zeta potential, are commonly encountered in alkaline electrolytes. This negative zeta potential can enhance particle incorporation into the coating, as the substrate and its oxide layer act as an anode during electro-oxidation, carrying positive pulses under alternating current (AC) conditions. Furthermore, the absolute value of the zeta potential increases with the electrolyte’s pH, facilitating better particle dispersion and interaction (Figure 3) [222].

The size and density of particles also significantly impact their stability within the PEO electrolyte. Most studies focus on particles smaller than 10 µm, as smaller particles are less prone to settling. Techniques such as mechanical agitation, gas bubbling, electrolyte pumping, and ultrasonic agitation are commonly employed to prevent particle settling and agglomeration. Additionally, surfactants like PTFE, MnO_2_, and NiO are sometimes added to enhance particle dispersion stability [223].

Another method to improve particle dispersion involves the use of in situ ash suspensions during electrolyte preparation. These suspensions are often synthesized using organic solvents like ethanol or specific complexing agents. While effective in producing stable sols, these additives can act as undesirable components in the electrolyte and may adversely affect the quality of the resulting PEO coatings [210].

Table 1 summarizes various particles introduced into the PEO electrolyte to enhance coating properties, including oxidation resistance, durability, and additional functionalities such as biocompatibility, antibacterial properties, ferromagnetic behavior, and catalytic activity [212]. These findings highlight the potential of particle-enhanced electrolytes to tailor PEO coatings for diverse applications.

Many bioactive Ca-P-containing PEO coatings have been successfully produced using electrolytes containing soluble calcium and phosphate salts, such as calcium acetate and sodium ortho- or hydrogen phosphates [244]. These electrolytes behave as suspensions due to the precipitation of calcium phosphates or hydrogen phosphates during the reaction, effectively functioning as particle-containing electrolytes. The phase composition of the coatings formed in these suspensions depends strongly on the PEO regime. For instance, studies by Matykina et al. [245] and Whiteside et al. [246] demonstrated that PEO conducted under direct current resulted in coatings containing anatase and rutile, with calcium and phosphorus present as amorphous phases. In contrast, the use of DC voltage [247] or bipolar regimes [248] promoted the formation of crystalline Ca-P-containing phases such as apatite, hydroxyapatite, and calcium titanate. This crystallinity is attributed to the high peak currents and localized temperatures reached during the positive pulses of the constant voltage mode.

Regarding corrosion resistance, various particles, including ZrO_2_, TiO_2_, and CeO_2_, have been incorporated into PEO coatings on magnesium and its alloys to enhance their performance. However, the results have been inconsistent, with the observed improvements often attributed to the formation of stable phases (reactive inclusion) or the inert incorporation of chemically stable particles [249,250,251]. For example, adding ZrO_2_ particles (200–400 nm) has been shown to significantly reduce the corrosion current density of coated magnesium alloys, from 7.27 × 10^−7^ A/cm^2^ to 7.03 × 10^−8^ A/cm^2^. This reduction corresponds to increased polarization resistance and a shift in corrosion potential to more positive values. Furthermore, salt spray tests confirmed that ZrO_2_ particles effectively minimized pitting propagation on PEO-coated substrates [252]. Reactive inclusion of ZrO_2_ particles via ash suspensions has also been found to enhance corrosion resistance, halving the corrosion current density and shifting the corrosion potential from −1.50 V to −1.22 V versus SCE [252].

**Figure 4:**
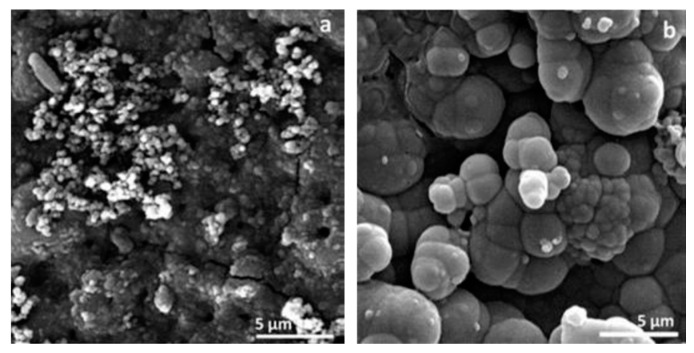
Apatite-forming ability of (**a**) PEO and (**b**) PEO incorporated with particles after immersion in SBF for 3 days. Reproduced with permission [256].

The incorporation of particles, whether inert or reactive, often results in denser or thicker coatings that exhibit improved barrier properties [207,253]. However, excessive particle concentrations in the electrolyte can increase coating porosity, reducing its protective capabilities [254]. In some cases, inert particles can serve as containers for corrosion inhibitors, providing self-healing functionality. For instance, Mingo et al. [255] used halloysite nanotubes loaded with benzotriazole to produce inhibitor-containing PEO coatings capable of responding to pH changes for active corrosion protection. Similarly, hydroxyapatite (HA) particles not only improved corrosion resistance but also imparted excellent apatite-forming capabilities to coatings on magnesium alloys, significantly increasing the amount of apatite formed after three days of immersion compared to pure PEO coatings (Figure 4) [256].

Despite these advancements, challenges remain. The coating production process can be time consuming, and in some cases, particle inclusion can negatively affect corrosion protection. For example, the addition of SiO_2_ nanoparticles improved short-term resistance but reduced long-term stability, as coatings with SiO_2_ particles showed higher degradation rates, eventually equaling the performance of coatings without particles [257,258]. Particle size also plays a critical role, with smaller particles inducing greater variability in corrosion properties. Wang et al. [259] reported that PEO coatings formed in electrolytes containing 5–10 vol.% TiO_2_ exhibited worse corrosion resistance after prolonged immersion in simulated body fluid (SBF), likely due to an increased amount of amorphous material resulting from TiO_2_ inclusion.

Infection risks associated with superficial implants can be mitigated through surface modifications, which play a crucial role in creating biocompatible surfaces for materials like titanium, tantalum, zirconium, and aluminum. Electrochemical methods such as anodic spark sputtering form oxides and unique surface topographies. For instance, titanium naturally forms a chemically stable oxide layer in air, but when exposed to physiological environments under mechanical stress, titanium ions may migrate into tissues, potentially causing allergic reactions, peri-implantitis, or hypergranulation [260,261,262,263,264]. Plasma electrolytic oxidation (PEO) addresses these issues by modifying the chemical composition, structure, and thickness of TiO_2_ layers, reducing ion migration and enhancing surface stability.

PEO continues beyond anodization, forming ceramic coatings with unique properties on magnesium, aluminum, and titanium alloys. Variants like Ticer and TiUnite have been successfully used in clinical practice for their enhanced wear and corrosion resistance, biocompatibility, and thermal stability [265,266,267,268]. The PEO process involves anodization followed by dielectric breakdown and microplasma formation, enabling the incorporation of elements such as calcium (Ca) and phosphorus (P) from the electrolyte. These ceramic oxide layers exhibit high adhesion strength (up to 26 MPa) and a porous structure that supports bioactive functionality [269,270,271].

Electrolyte composition and anode voltage significantly influence surface properties. Incorporating bioactive ions like Ca and P into PEO coatings improves osseointegration by forming apatite and hydroxyapatite, closely mimicking bone tissue [246,272,273]. Enhanced corrosion resistance is achieved by adding particles like ZrO_2_, TiO_2_, or SiO_2_, which form stable phases or inert inclusions within the oxide layer [274,275,276]. These coatings improve surface–bone interaction, as shown by torque tests and histological studies, with Ca/P coatings demonstrating high mechanical stability and excellent bone growth [277,278,279].

Studies by Ishizawa et al. [280] highlighted the mechanical and biological advantages of Ca-P-containing PEO coatings, showing that hydroxyapatite (HA) and oxide layers promote osteoid formation and bone adhesion. Implants with PEO coatings exhibited significantly higher removal torque values compared to untreated titanium, indicating superior bone integration. Moreover, rabbit and dog studies have demonstrated that PEO surfaces doped with factors like rhBMP-2 significantly stimulate bone growth [281,282,283,284,285].

Recent developments in combining zirconium and titanium for implant surfaces leverage zirconium’s biocompatibility and titanium’s mechanical strength. Shin et al. [286] demonstrated that PEO coatings containing tetragonal ZrO_2_ improve osteoblast proliferation and biomimetic apatite formation. Modifications such as alkali treatments, plasma spraying, sol-gel techniques, and PEO have further enhanced ZrO_2_-based surfaces’ bioactivity, wear resistance, and corrosion resistance [287,288,289,290,291,292].

HA coatings, known for their composition resembling human bone, have been extensively studied for their osteoconductive properties. Nanosized HA particles improve surface bioactivity and cell proliferation more effectively than microsized particles, enhancing apatite deposition during immersion in simulated body fluid (SBF) [293,294,295,296,297]. HA can be combined with ions like magnesium, zinc, and silicon to improve bioactivity and mechanical properties further. For example, Zn-doped HA supports nucleic acid metabolism and protein synthesis, promoting bone regeneration [298,299,300,301,302,303].

PEO has also been applied to emerging titanium alloys like Ti-3Zr-2Sn-3Mo-25Nb (TLM) and Ti-13Nb-13Zr, demonstrating improved biocompatibility and osteoconduction. Coatings containing β-tricalcium phosphate and anatase TiO_2_ enhance osseointegration by providing open structures conducive to cell adhesion and proliferation [283,304,305]. Despite these advancements, challenges remain, including optimizing electrolytes, ensuring long-term surface stability, achieving antibacterial properties, and scaling up mass production of implants with PEO coatings. Further research is needed to understand cell responses and osseointegration processes over extended periods.

## 5. Coating Thickness

M. A. Faghihi Sani et al. reported that a coating thickness of 2.3 µm was achieved after a PEO process conducted at a current density of 0.212 A/cm^2^ in an electrolyte containing 0.222 mol/L calcium acetate and 0.040 mol/L calcium glycerophosphate [265]. Similarly, S. M. et al. utilized a mixture of 5 g of hydroxyapatite (HA) powder, 10 mL of ethylene glycol, and 5 mL of triethanolamine to prepare a stable dispersion, ensuring effective nanoparticle inclusion within the coating structure. Additionally, 5 g of trisodium orthophosphate was introduced into the dispersion as an electrolyte component.

**Figure 5:**
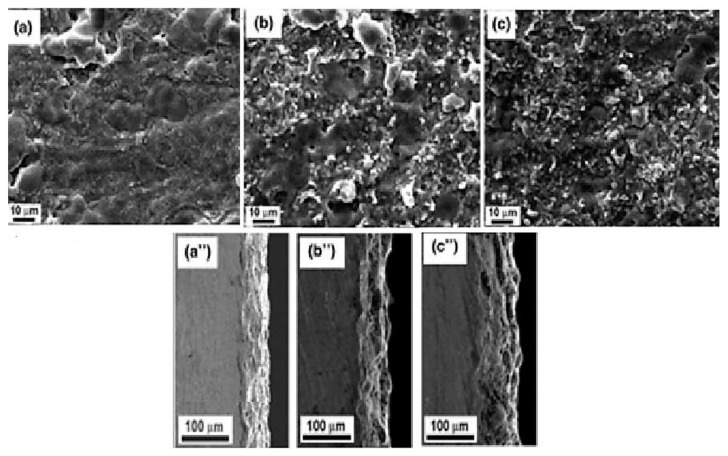
SEM top and cross-sectional view and EDS spectroscopy of the PEO fabricated at different times ((**a**,**a”**) 2, (**b**,**b”**) 4, and (**c**,**c”**) 6 min). Reproduced with permission [306].

The PEO process was carried out at a frequency of 50 Hz using a constant current density of 150 mA/cm^2^ for 6 min, employing a direct current (DC) power source at 900 V and 15 A. During this process, the thickness of the nanodiamond (ND) groups increased to 2 μm within the first 2 min. With the application of PEO combined with electrophoretic deposition (EPD), the coating thickness values further increased to 58 and 75 µm, as illustrated in Figure 5 [306].

Increasing the concentration of electrolytes during the PEO process has been shown to enhance the thickness of the formed structure. The growth rate of the layer was more pronounced with increased calcium acetate concentrations (10.1 µm in 6 min) compared to β-glycerol phosphate (6.6 µm in 3 min) [307]. Typical PEO solutions often contain hydroxyapatite and calcium acetate phases, ensuring the biocompatibility of the resulting structures. The hydroxyapatite layer formed during the process is initially amorphous and rapidly solidifies.

The adhesion strength between the oxide film and the substrate generally increases with longer PEO processing times [308,309,310,311]. While the growth rate accelerates slightly over the first 30 min, the coating reaches approximately 49 µm in thickness during this period. At 120 min, maximum coating thickness (64 µm) and pore size (8 µm) are achieved [312]. However, as noted by M.S. Kim et al., element concentration does not directly affect coating thickness [313], though higher electrolyte concentrations can decrease adhesive strength [314,315]. Additionally, slower growth rates are observed when the voltage drops during the process [316].

Surface modifications achieved through electrochemical methods, such as anodic spark deposition (ASD), play a critical role in developing biocompatible surfaces for various materials. Techniques like ASD, micro-arc oxidation (MAO), plasma electrolytic oxidation (PEO), and dielectric breakdown have been widely used to form ceramic layers on anode metal substrates. These surfaces, including clinically applied variants like Ticer and TiUnite, have demonstrated long-term success in enhancing implant performance [317,318].

**Figure 6:**
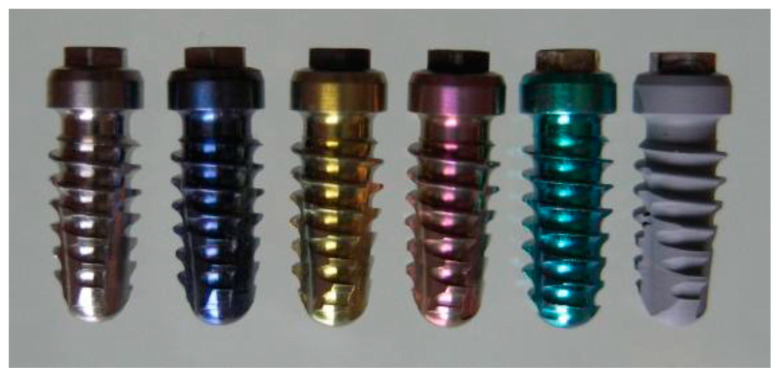
Uncoated Ti cp with colorless native oxide film, from blue to green, the electric-potential-dependent anodic pre-spark films, and the gray surface of an anodic spark-discharge-generated coating (left to right). Reproduced with permission [319].

Base metals such as titanium, tantalum, zirconium, and aluminum spontaneously form oxide layers in acidic environments. This property is exploited during anodic spark deposition to create oxides with specific topographies. For titanium, a thin TiO_2_ layer forms within nanoseconds, providing a passivating oxide film [319,320]. Spark discharge anodic oxidation (ASD) further modifies the TiO_2_ layer’s chemical composition, structure, and thickness. This process begins with “pre-spark anodizing”, where a thin anodic Ti-oxide film (approximately 100 nm) develops on the titanium surface, often displaying distinct color variations (Figure 6) [317,321].

**Figure 7:**
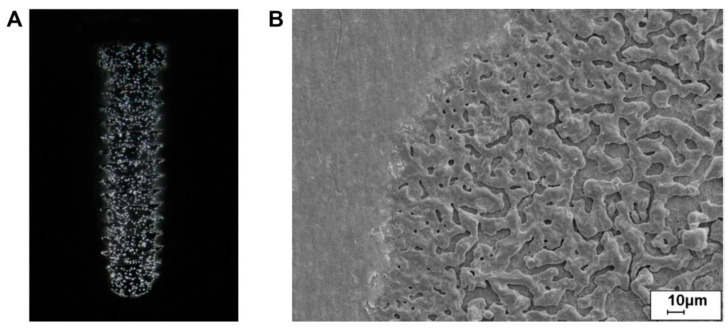
(**A**) Visible micro plasma sparking at a Ti dental implant as an anode during ASD. (**B**) Anodic pre-spark conversion film (**left**) and the initial state of the ASD process of a Ti surface with first spark-discharge-generated molten oxide traces (**right**) because of the spark avalanches. Reproduced with permission [317].

During the anodization phase that precedes sparking, the metal–oxide and oxide–electrolyte interfaces are progressively replaced by the metal–electrolyte interface. This transition results in potentiodynamic anodizing, characterized by a gradual damping of the exponential drop in anode current. Subsequently, a galvanostatic increase in electric potential induces dielectric breakdowns, visible as microplasma sparking on the anode surface (Figure 7A), and initiates the formation of a new ceramic coating [322,323].

Further increases in the anodic potential lead to vertical growth of ion-conducting oxide films formed on titanium metal surfaces. Ions from the metal or electrolyte side migrate into the oxide phase, contributing to additional film growth at the interface (Figure 7B) [317].

The dielectric breakdowns observed during anodization are explained by the appearance of charge carrier avalanches through the oxide film on the anode. As a result, the coatings incorporate compounds derived from both the electrolyte and the anode material. For instance, elements like calcium (Ca) or phosphorus (P) can be integrated into the oxide layer depending on the composition of the electrolyte and the specific coating conditions [324].

**Figure 8:**
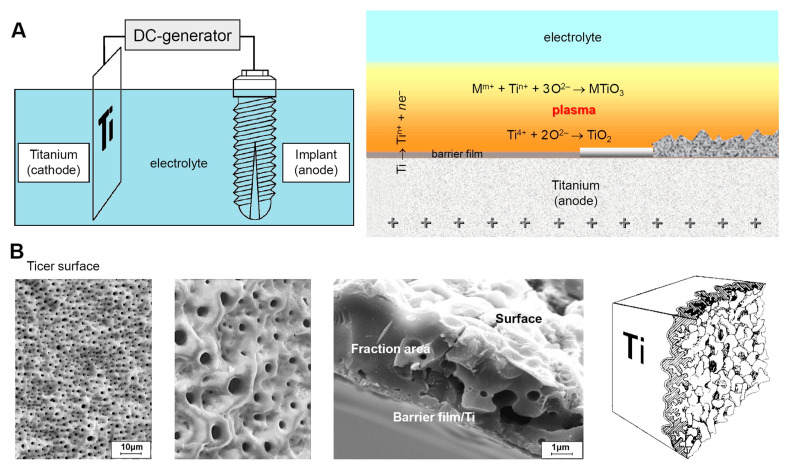
Preparation of Ticer using anodic spark deposition: (**A**) electrolyte cell and ASD process on anode; (**B**) surface upon treatment (SEM and schematic representation). Reproduced with permission [317].

As the oxide film continues to grow under a constant anodic potential during breakdown, the voltage across the film eventually becomes insufficient to sustain the critical electrical breakdown field. This self-limiting mechanism halts the anodic spark deposition (ASD) process, with the film thickness determining the termination point. The anodization of pure titanium produces surfaces with varying oxide layer thicknesses depending on the applied voltage and anodization duration (Figure 8) [317,325].

Different electrolytes, such as sulfuric acid, phosphoric acid, acetic acid, sodium hydroxide, or calcium hydroxide, are commonly used in anodic spark deposition (ASD) to achieve varying oxide layer thicknesses. The formation stress on the anode is higher in acidic electrolytes compared to alkaline ones [326]. Increasing the concentration and temperature of the electrolyte reduces the voltage required for oxide layer formation. Conversely, increasing the current density, as well as the ratio of the anode surface area to the cathode, leads to higher voltage and layer thickness.

ASD coatings are predominantly X-ray amorphous, with anodic oxidation of pure titanium producing mainly anatase. Applying higher voltages during the spark discharge phase can result in thicker oxide layers comprising a mixture of rutile and anatase TiO_2_ modifications [327]. Anatase crystallizes in a tetragonal system and is colorless in pure form, while rutile, the most stable TiO_2_ phase, also has a tetragonal crystal structure. Brookite, another TiO_2_ modification, crystallizes in an orthorhombic system. Anatase can transition to rutile at approximately 915 °C.

The ceramic oxide layers formed during ASD offer unique advantages, including a highly porous structure (Figure 8B), strong adhesion to the substrate (up to 26 MPa), modifiable chemical composition, and long-term stability. These properties have been successfully utilized for surface modifications of titanium implants, ensuring improved osseointegration [328,329].

The choice of electrolyte significantly influences the physical and chemical properties of the oxide layer. Residual electrolytes often remain in the pores of the coating, impacting its performance. The oxide layer can also be doped with bioactive substances, such as calcium or phosphorus ions, enhancing the bioactivity of the implant and promoting bone integration. For example, Ishizawa et al. investigated TiO_2_ layers formed in electrolytes containing calcium and phosphate ions. An oxide layer enriched with hydroxyapatite (HA) was sintered under high pressure (300 °C), resulting in implants with varying layer thicknesses and HA concentrations. Implants were tested in rabbit femurs for 8 weeks, showing similar removal torques (~20 MPa) for HA-modified surfaces, whereas untreated titanium and non-thermally treated oxide layers required significantly lower removal forces (2 and 15 MPa, respectively) [330]. Ruptures occurred within newly formed bone rather than at the interface between the implant and the oxide layer or between the oxide layer and HA.

**Figure 9:**
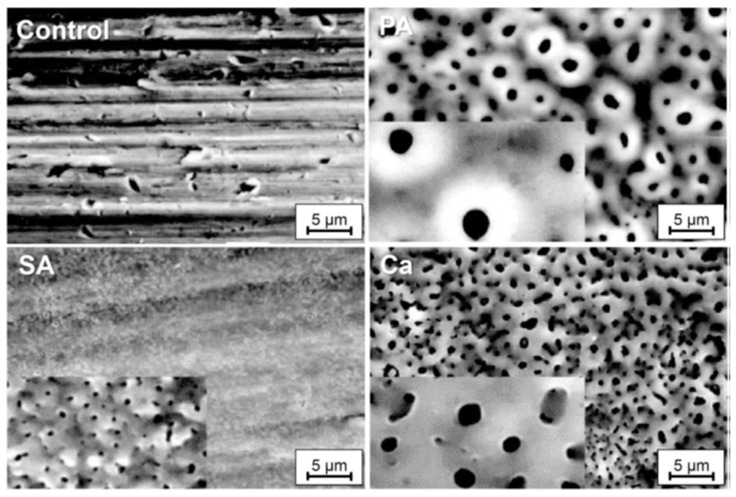
SEM of non-porous (control) and porous surfaces (PA, SA, and Ca). Reprinted and adapted from [331], Copyright 2003, with permission from Elsevier.

Further studies have examined bone formation on surfaces obtained via ASD using different electrolytes, including sulfuric acid (SA), calcium ion solutions (Ca), and phosphoric acid (PA) (Figure 9) [331]. Implants prepared with SA and Ca electrolytes exhibited significantly higher removal torque 6 weeks post implantation in rabbits compared to those prepared with PA. SA- and Ca-treated surfaces also yielded oxide layers approximately 1100 nm thick, whereas control implants had only 17 nm thick layers. Histomorphometric analyses revealed the highest levels of bone integration for HA-coated surfaces, with evidence suggesting a biochemical interaction between the implant surface and bone tissue in PA- and Ca-treated samples.

Narayanan et al. proposed that the porous structure of the oxide layer allows it to absorb liquids and ions from the surrounding bone tissue, facilitating interaction and osseointegration [332]. In a study by Leknes et al., 36 coated screw implants doped with varying concentrations of recombinant bone morphogenetic protein-2 (rhBMP-2) were implanted in 12 dogs [333]. The authors concluded that TiUnite surfaces acted as effective rhBMP-2 carriers, significantly stimulating bone growth. An overview of protein-coated surfaces is presented in Table 2 [317].

Hilbig et al. examined the effects of coated and uncoated surfaces with bone sialoprotein (BSP), collagen, and fibronectin on human maxillary bone development in vitro. These surfaces included commercially pure titanium (Ti cp) and Ticer coatings [335]. Similarly, Graph et al. investigated the in vitro behavior of bone cells on Ti cp and Ticer coated with BSP, type I collagen, and hydroxyapatite, mimicking the primary organic and inorganic components of bone [336].

Electrochemical anodizing methods have emerged as a promising approach for producing nanotubular and nanoporous modifications, which hold significant potential for enhancing dental and orthopedic implants. The hierarchical structure of bone, particularly at the nanometer scale, aligns well with the structural features of these materials, making this process highly relevant in orthopedic research [337].

Expanding research has revealed the advantages of TiO_2_ nanotubes in improving the surfaces of orthopedic implants. A crucial factor in preparing self-assembling TiO_2_ nanoporous structures is the fluoride ion concentration in the electrolyte. The fluoride concentration, pH value, and anodization duration are critical parameters in forming metal oxide (MOx) nanotubes [338,339]. Anodization for creating nanotubular layers is typically performed by ramping up the potential and maintaining a constant voltage, ranging from 1–30 V in aqueous electrolytes to 5–150 V in non-aqueous electrolytes, with fluoride anion concentrations between 0.05 and 0.5 M [340].

**Figure 10:**
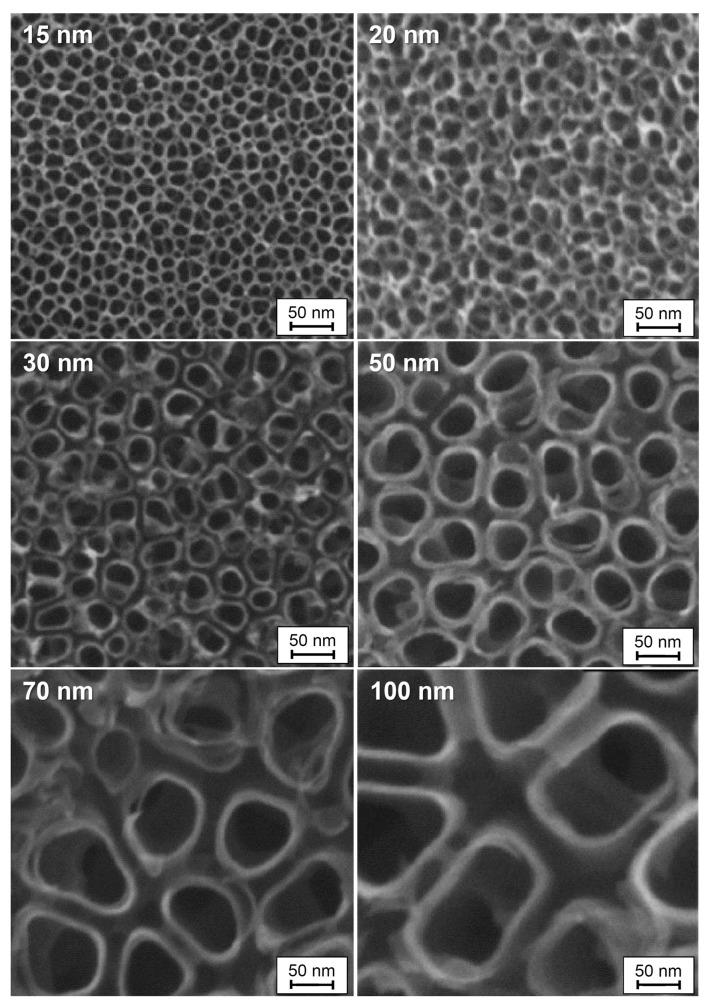
The surface of layers of self-aligned TiO_2_ nanotubes have different pore sizes (between 15 and 100 nm). Self-assembled layers of vertically oriented TiO_2_ nanotubes were generated by anodizing titanium sheet. Reproduced with permission [105,341].

The diameter and three-dimensional tubular nanostructures of TiO_2_ nanotubes are directly influenced by the applied anode potential. Both in vitro and in vivo studies have demonstrated the biomedical potential of these oxide nanotubes. For instance, experiments conducted in vitro and with mini-pigs revealed that TiO_2_ nanotubes with diameters of approximately 15 nm (Figure 10) significantly enhanced cell adhesion, proliferation, and differentiation. In contrast, nanotubes with diameters exceeding 50 nm induced programmed cell death, underscoring the importance of precise dimensional control in biomedical applications [105,317,341].

The same authors reported that TiO_2_ nanotubes with pore diameters exceeding 50 nm can benefit certain cellular functions. Additionally, Hu et al. observed that 100 nm TiO_2_ nanotubes significantly enhanced osseointegration both in vitro and in vivo (rabbit tibias) compared to controls and microtopographic surfaces [342]. These TiO_2_ nanotube-coated implants demonstrated remarkable improvements in new bone formation, bone-related gene expression, and bone remodeling compared to flat surfaces [343].

Nanotubes also offer the potential to be loaded with antibacterial agents to prevent infections [344]. For example, silver inclusions can be incorporated into the nanotubes to enhance antimicrobial properties. However, the number of clinical studies on nanotube applications remains limited, highlighting the need for further research [345,346].

Nanostructured surfaces on titanium implants have also been obtained through anodic oxidation techniques [347]. In parallel, zirconium’s mechanical properties (strength comparable to stainless steel), excellent biocompatibility, and color similarity to natural teeth have driven interest in combining zirconium and titanium to improve implant surfaces [348]. However, challenges remain. For instance, zirconium oxide implant bodies coated with titanium oxide do not fully address issues related to zirconium oxide’s mechanical stability or titanium oxide’s potential toxicity [349].

**Figure 11:**
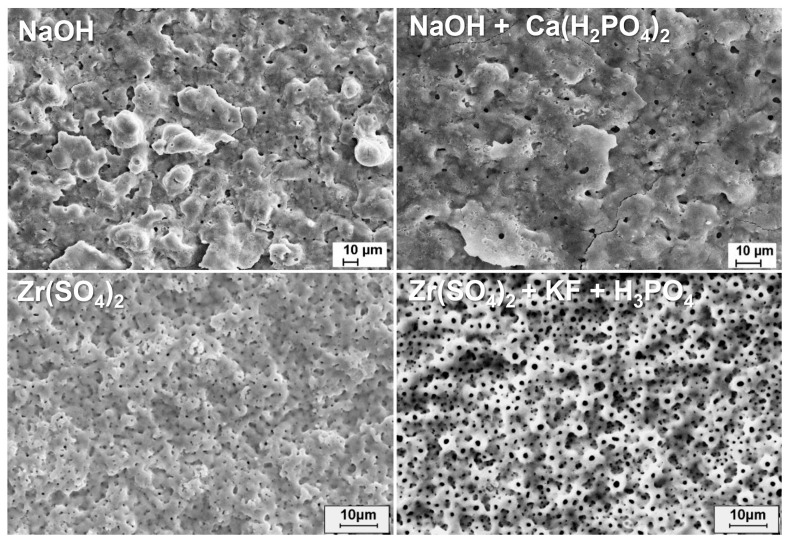
Some newly developed titanium-based implant surfaces prepared using different electrolyte systems (**upper**—two white surfaces (Ticer white); **lower**—two zirconia-coated Ti cp surfaces). Reproduced with permission [317,350].

A promising alternative involves implants where the body is titanium-based, and the surface coating is zirconium dioxide. Anodic plasma electrochemical oxidation has been employed to create novel surfaces using varying concentrations of Zr(SO_4_)_2_ in aqueous electrolytes. Further, this electrolyte system, supplemented with KF and/or H_3_PO_4_, was used to fabricate surfaces with enhanced features (Figure 11) [317,350].

These surfaces exhibited in vitro effects on osteoblasts similar to those observed with Ticer, particularly enhancing the rate of osteoblast differentiation compared to smooth surfaces. The surfaces also positively influenced osteoblast morphology, including changes in cell shape and the formation of cell clusters. Notably, titanium’s mechanical stability and zirconium’s biological compatibility were preserved in these composite coatings.

The interaction between the physicochemical properties of implant surfaces and surrounding tissue plays a critical role in osseointegration. It can be further enhanced by targeted ionization or growth factors. When tissue comes into contact with the implant surface, an ion-protein exchange occurs, influencing the integration process. The bond strength between the implant material and bone tissue is commonly evaluated by measuring the torque required to remove the implant. Exceptionally high torque values have been achieved on surfaces doped with phosphate and calcium ions, reflecting superior bonding and osseointegration. Numerous in vitro studies using animal and human osteoblast cell cultures have demonstrated that anodically oxidized surfaces with various additives significantly enhance cell maturation and differentiation.

**Figure 12:**
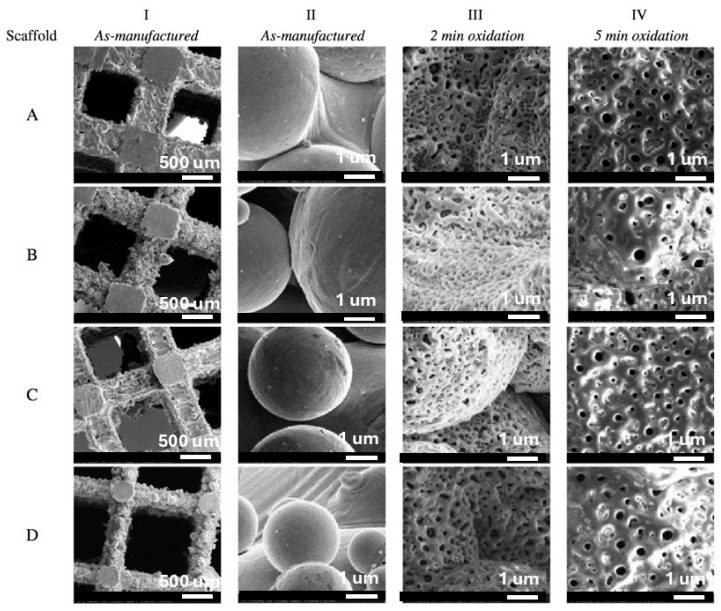
SEM micrographs of as-manufactured (I, II), 2 min (III), and 5 min (IV) PEO-treated scaffolds (**A**–**D**). Reproduced with permission [263].

The development of advanced manufacturing technologies has brought metal porous biomaterials into focus, enabling the creation of more complex structures. At the same time, biofunctional surfaces on metal implants are essential for improving biomechanical performance. Scanning electron microscopy (SEM) analysis revealed the formation of pores on scaffold surfaces treated with PEO, with oxide layer thicknesses of 4.85 ± 0.36 µm after 2 min of processing and 9.04 ± 2.27 µm after 5 min (Figure 12) [263].

Oxidation during the plasma electrolytic oxidation (PEO) process does not significantly affect most mechanical properties of titanium implants, such as maximum allowable stress, yield strength, stress plateau, and energy absorption. Still, it does have a notable impact on the elastic modulus. Orthopedic scaffolds are crucial in mimicking bone structures and supporting new tissue formation in bone tissue engineering. These scaffolds must induce osseointegration, be biocompatible, and possess mechanical properties compatible with surrounding bone tissue [351,352]. One of the primary challenges in repairing bone defects is fabricating scaffolds with biomechanical properties similar to natural bone [353].

Due to their superior strength, metal structures have become the leading choice for orthopedic and dental implants [354]. However, stress shielding remains a significant issue with titanium alloy implants. This phenomenon arises from the mismatch between the elasticity modulus of the implant and bone, leading to micromovements in the peri-implant zone. Porous implants effectively address this issue by reducing the elasticity modulus to values closer to that of bone tissue, thereby balancing load transfer and minimizing stress shielding. Additionally, interconnected hollow pores provide essential pathways for nutrient delivery, vascularization, and implant fixation [355,356].

The 3D printing method offers advanced capabilities for manufacturing porous metal implant frameworks. This technology allows for the precise control of porosity, morphology, and pore size, enabling the creation of implants with predictable mechanical properties tailored for specific biomedical applications [357,358].

Studies on bulk titanium alloys indicate that PEO layers can influence fatigue behavior [359,360]. However, the effects of PEO layers on the mechanical behavior of additively manufactured titanium frameworks remain unexplored. Research involving selective laser melting (SLM)-fabricated scaffolds with porosities ranging from 13% to 37% demonstrated the formation of specific PEO micropores on all surfaces after oxidation using calcium acetate and glycerophosphate electrolytes at a current density of 20 A/cm^2^ for 2 and 5 min. SEM analysis revealed that longer oxidation times reduced pore density but resulted in larger, more uniformly distributed pores. Energy-dispersive X-ray spectroscopy confirmed the incorporation of calcium and phosphorus from the electrolyte into the oxide structure, with the Ca/P atomic ratio increasing from 1 to 1.5 after 5 min of oxidation.

The thickness of the oxide layer increased from 4.9 ± 0.4 μm to 9 ± 2.3 μm with longer oxidation times. While the 2-min oxidation process did not affect the mechanical properties of the scaffolds, the 5-min process resulted in a reduction of up to 30%, particularly in high-density frameworks. Fatigue characteristics, however, remained unchanged. Analytical and numerical studies indicated that the modulus of elasticity of additively manufactured porous implants is lower than predicted, likely due to micropores in the structure. Nevertheless, numerical and experimental values for elastic modulus and fracture stress were closely aligned [263].

**Figure 13:**
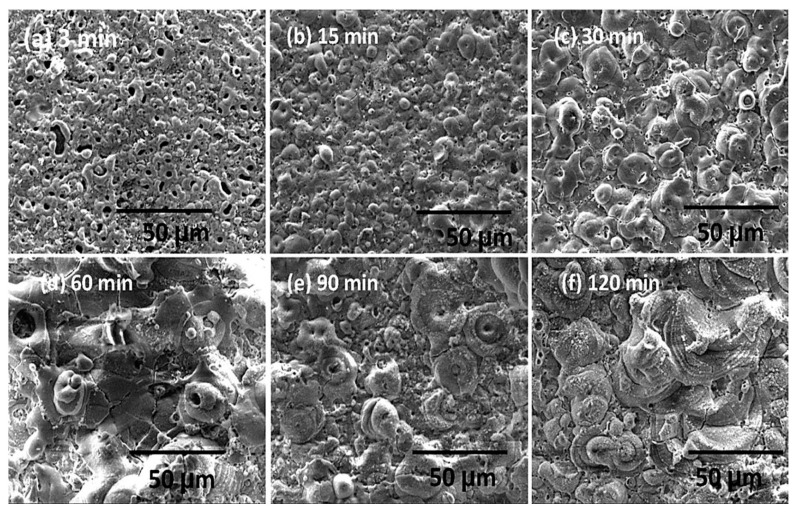
SEM images (**a**–**f**) of the oxide layer on AJ62 during various PEO (MAO) treatment times. Reproduced with permission [362].

PEO conducted in a solution containing 10 g of sodium aluminate (Na_2_Al_2_O_4_) and 1 g of potassium hydroxide (KOH) using a bipolar current mode resulted in a porous structure forming within the first 3 min of a 120-min process. Figure 13 illustrates the outer layer’s porous structure and the dense inner layer, which adhered well to the substrate [361].

Voltage control during micro-arc oxidation (MAO) influences pore formation. Lower voltages tend to produce uniformly distributed spherical micropores, while higher voltages increase pore size, providing a tunable approach for tailoring surface characteristics [362].

**Figure 14:**
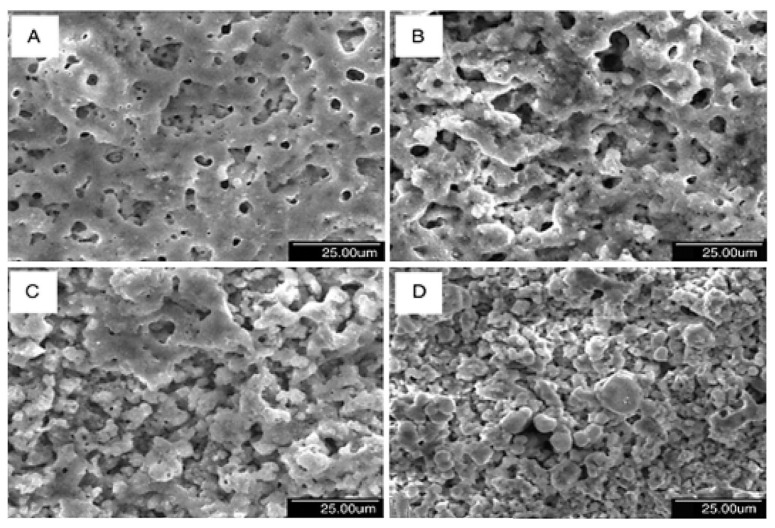
SEM view of the structured layer at 480 V treated for (**A**) 1.5, (**B**) 3, (**C**) 10, and (**D**) 20 min via the oxidation process (MAO). Reproduced with permission [363].

J. Sun et al. observed that the phase peaks of rutile (R-TiO_2_), perovskite (CaTiO_3_), and α-tricalcium phosphate (α-TCP) were detectable across all applied voltages in their experiments. The study utilized an electrolyte containing 0.02 M β-glycerophosphate and 0.2 M calcium acetate, with processing conducted at constant voltages ranging from 400 to 480 V for durations of 1.5 to 20 min. The characteristic pore structure disappeared when the voltage and time were increased to 480 V and 20 min, respectively. This was attributed to the deposition of hydroxyapatite (HAp) and calcium carbonate (CaCO_3_), which completely covered the TiO_2_ film. Notably, the pore structure was visible only at voltages up to 430 V (as shown in Figure 14) [363].

M. Montazeri et al. reported that rutile and brookite phases were present at all applied voltages during the micro-arc oxidation (MAO) process. When the voltage increased to higher values, such as 500 V, most of the pores on the surface disappeared due to the coating of the hydroxyapatite (HAp) phase by a titanium oxide film [364]. Similarly, S. Abbasi et al. observed that with an increasing duration at a constant voltage during the MAO process, the diameter of the resulting pores reached up to 280 nm. Additionally, increasing the calcium acetate concentration from 5 to 10 g/L enhanced the density of the pores, attributed to the intensification of electric discharges on the surface caused by the higher electrolyte concentration [365]. Conversely, another study reported that an increased concentration of β-glycerol phosphate led to a reduction in pore density within the created layer [366].

**Figure 15:**
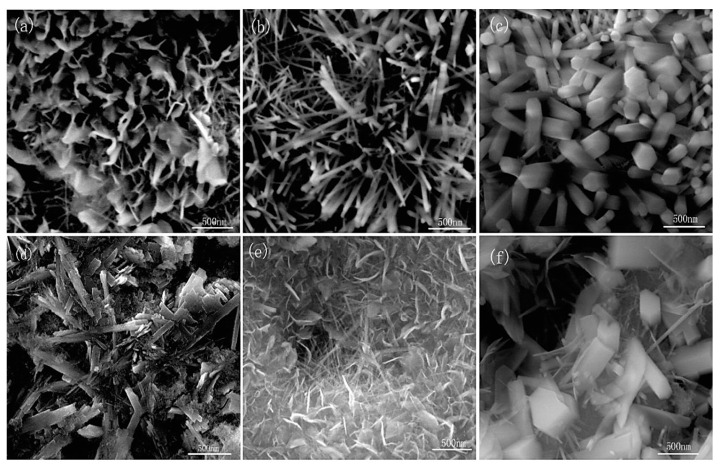
SEM images of different HA structures after MAO hydrothermal treatment for 6 h ((**a**) 150 °C, (**b**) 200 °C, and (**c**) 250 °C, (**d**) 250 °C—6 h in 100 mL solution, (**e**) 250 °C—6 h in 400 mL solution; (**f**) 250 °C—12 h in 200 mL solution). Reproduced with permission [367].

The influence of heating temperature variations and immersion duration was also investigated. The concentration and activity of copper (Cu) and phosphorus (P) were identified as key factors in the formation of nanocrystalline (ND) structures. High heating temperatures combined with prolonged immersion in a 200 mL solution led to the formation of a stable film on the surface of the titanium alloy. Figure 15 illustrates the hexagonal needle-like columns and brittle hydroxyapatite (HA) crystal structures formed under these conditions [367].

During the PEO process, the size and distribution of pores change over time. Initially, the pore structures are uniformly distributed within 10 min, but the structures become randomly separated with prolonged processing up to 120 min [312]. Crack formation may occur during the PEO process due to thermal stress generated at the surface [368]. Kim et al. noted that the concentration of CaCl_2_ in the electrolyte significantly influences the morphology of the biofilm formed during the PEO process. Their experiments involved titanium-based samples immersed in an aqueous solution of calcium chloride and potassium phosphate in deionized water at 50 °C.

Research into the PEO process for zirconium (Zr) alloys has focused on understanding oxide film growth kinetics and its phase transformations. However, the exact mechanisms of oxide formation and phase transitions on zirconium remain only partially understood [369,370,371]. In an attempt to explore these mechanisms, commercially pure zirconium surfaces were subjected to PEO in silicate-based electrolytes for varying durations (5, 10, 20, 30, 45, 60, 90, and 120 min). The electrolyte consisted of 12 g/L sodium silicate (Na_2_SiO_3_) and 2 g/L potassium hydroxide (KOH) dissolved in distilled water, with a pH of 12.9. The PEO process was conducted using an asymmetric AC power supply (50 Hz) with a maximum power of 100 kVA. The coating formation voltages were fixed at 480 V (positive peak) and 120 V (negative peak), with a constant current density of approximately 0.25 A/cm^2^.

The process included agitation of the electrolyte with compressed air and temperature control, maintained at 23 °C ± 3 through cold water circulation around the electrolyte cell. After each processing step, the zirconium samples were cleaned in distilled water, ultrasonicated in ethanol for 5 min, and dried with warm air. Coating thickness was measured using an eddy current instrument (Fischer), with 10 measurements taken from various locations to ensure accuracy.

**Figure 16:**
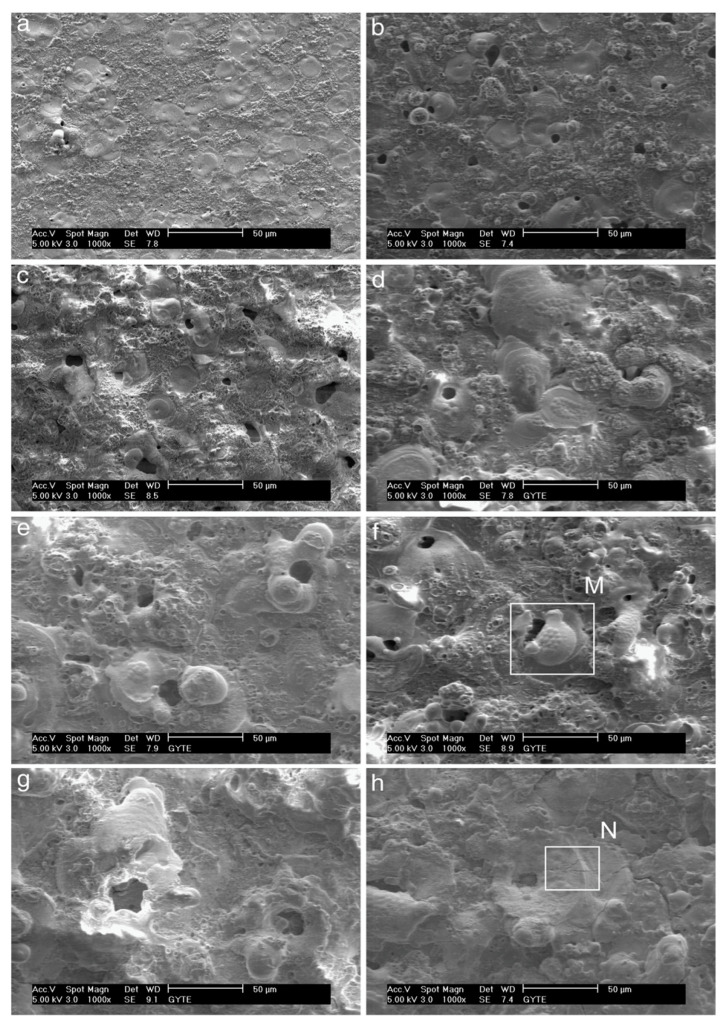
The surface SEM micrographs of PEO-coated pure zirconium for the period of (**a**) 5 min, (**b**) 10, (**c**) 20, (**d**) 30, (**e**) 45, (**f**) 60, (**g**) 90, and (**h**) 120 min, successively. Reproduced with permission [370].

SEM analysis of PEO-coated zirconium samples revealed significant changes in surface morphology over time. At the initial 5-min stage, the coating surface exhibited a smooth “pancake-like” appearance with an approximate diameter of 10–20 µm. These features were randomly distributed and contained uniform micropores less than 1 µm in diameter at their centers (Figure 16a) [370].

The number of micropores on the surface decreases, and their size noticeably increases, reaching approximately 25 µm with extended PEO durations (Figure 16b–h). The pancake-like features observed during the initial stages of the process completely disappear when the duration exceeds 20 min. With longer processing times, material accumulation becomes more prominent around the pores, forming approximately spherical shapes or irregular conglomerates. These features exhibit a mixture of dense regions and random porosity across the surface.

**Figure 17:**
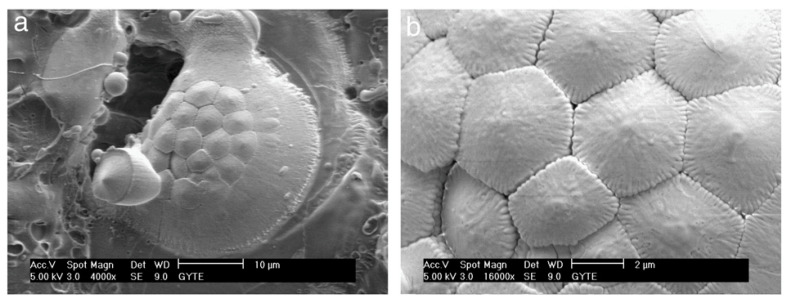
The high magnification of a typical SEM image of the equiaxed cluster is taken from Figure 16f (marked as “M”) for the process time of 60 min: (**a**) 4000× and (**b**) 16,000×. Reproduced with permission [370].

For processing times exceeding 20 min, spherical-like structures characteristic of equiaxed grains with straight boundaries begin to dominate the surface morphology (Figure 16d–h). At 60 min of processing, high-magnification SEM images (Figure 17a,b) reveal equiaxed clusters with grain sizes of approximately 3–4 µm. Additionally, Figure 17a shows evidence of a settling debris stream, with the marginal structure of the deposits resembling the boundaries of the equiaxed grains (Figure 17b).

**Figure 18:**
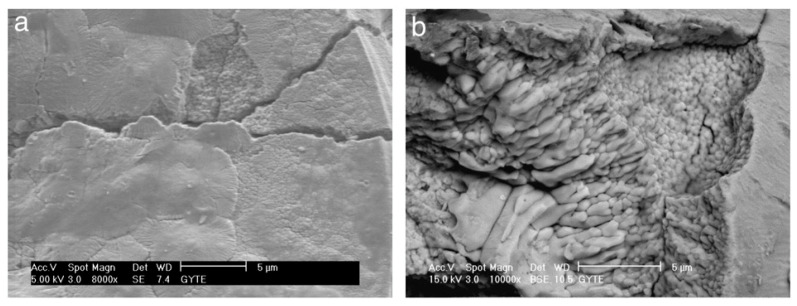
Typical SEM images from the surface: (**a**) the coating flakes off from the surface of Figure 16h (marked as “N”) with higher magnification; and (**b**) the presence of very fine equiaxed crystals just underneath the smooth regions around the plasma channel openings. Reproduced with permission [370].

Figure 18a provides a typical SEM image illustrating coating delamination from the surface. This delaminated region corresponds to a portion of Figure 16h (labeled “N”). At higher magnification, Figure 18b reveals the peeling of a smooth area of the coating surface. Beneath these smooth regions, fragile equiaxed crystals are visible. However, within the coating’s interior, these crystals become coarser and elongated, oriented outward.

Additionally, microcracks formed during the process are prominently visible in Figure 18b. These cracks are likely induced by thermal stresses and material redistribution during the PEO process [370].

The study revealed several key findings. Unique equiaxed clusters were identified on the surface of the PEO coating, with their characteristics evolving throughout the process on the zirconium surface. Radially grown plasma channels containing silicon crystals were observed on the cracked surfaces of the coating. Despite the modification process, the thickness of the monoclinic ZrO_2_ and tetragonal ZrO_2_ oxide film remained consistent, showing no significant changes.

## 6. Crystallinity Features

The crystal structure of hydroxyapatite (HA) can be developed through plasma electrolytic oxidation (PEO) [372]. In research conducted by W. H. Song et al., 0.04 mol/L β-glycerophosphate disodium salt pentahydrate and 0.4 mol/L calcium acetate monohydrate were utilized in the microarc oxidation process. This process was performed at constant voltages of 250, 300, 350, 400, and 450 V for a duration of 3 min. Their findings indicated that Na and P ions did not precipitate at voltages below 350 V, as the ions require divalent states to create a porous surface suitable for HA nanostructure formation.

At a voltage of 500 V, the crystalline structure became evident, with the oxide layer primarily comprising β-Ca_2_P_2_O_7_, CaTiO_3_, α-Ca_3_(PO_4_)_2_, and Ca_2_Ti_5_O_12_ [362]. In a separate study, M. A. Faghihi Sani et al. used an aqueous electrolyte containing calcium acetate and glycerophosphate with a Ca/P ratio of 6.8 during a PEO process for 4 min at a frequency of 100 Hz. Their SEM, XRD, and EDS analyses revealed that the crystallized HA layer had a stoichiometric Ca/P ratio of 1.67 [373].

M. Okido et al. investigated the impact of electrolyte pH on the surface properties of HAp by introducing sodium hydroxide (NaOH). Their findings showed that altering the pH, combined with the application of alternating current at a frequency of 60 Hz and a current of 30 A, influenced the Ca/P ratio of HAp. When the pH exceeded 6, needle-like and thin plate-like HAp layers were deposited, with a Ca/P ratio of 1.46—close to the value associated with the formation of CaA via electrochemical processes. In contrast, HAp crystallization did not occur at pH values below 7, as the reduction in OH^−^ ion concentration inhibited the deposition process [374].

**Figure 19:**
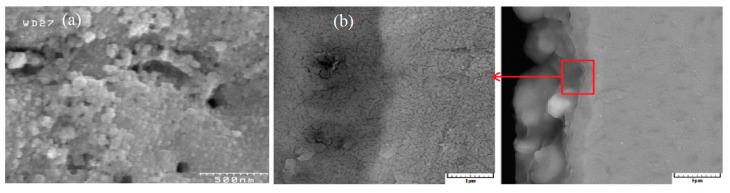
FESEM images of the layer grown in an electrolyte containing 5 g/L calcium acetate and 5 g/L β-glycerophosphate for 3 min (**a**) and cross-section of the two-layer interface (**b**). Reproduced with permission [307].

Research by S. Abbasi et al. confirmed that the coating produced during the PEO process consists of two distinct layers: a titanium base layer and an HA layer. The titanium layer was approximately 2 ± 0.2 µm thick, while the upper layer contained TiO_2_ and HA. This coating was achieved at 350 V over 3 min of microarc oxidation. Figure 19 displays SEM images from two stages of the microarc oxidation process, demonstrating a high level of surface bioactivity [307].

The largest HAp crystal size was achieved using a two-step MAO process. The first step employed 10 g/L trisodium phosphate as the electrolyte, while the second step combined β-glycerol phosphate and calcium acetate.

Durdu et al. conducted a PEO process on a titanium surface using calcium acetate and β-calcium glycerophosphate as the electrolyte. This process, performed at a current density of 0.123 A/cm^2^ and 30 kW (AC), was carried out for varying durations. The NA phase reached its peak structuring after 40 min, with the maximum quantity of NA observed at 120 min, indicating that crystal growth increased over time [312].

The limited growth of the surface layer can be attributed to the rapid cooling rate during HAp crystal nucleation within the electrolyte, resulting in nanometer-sized crystals and a layer thickness ranging between 1 and 100 nm. The process temperature was consistently maintained at 70 ± 3 °C, with constant voltage and solution circulation. Enhanced crystalline layer formation was observed with an increased concentration of calcium acetate, while β-glycerol phosphate did not yield similar results. S. Abbasi et al. reported that PEO treatment at 350 V for 3 min on a commercially clean titanium surface resulted in a growing ND structure within the 30–60 nm range [375].

Incorporating phosphate ions into titanium surfaces is a common strategy to promote bone tissue regeneration [376]. The PEO process generates oxide coatings with complex geometries, providing a robust bond between implants and bone. Given their positive effects on bone regeneration, recent research has focused on metal ions such as magnesium, zinc, strontium, and manganese. In particular, zinc ions (Zn) have been shown to enhance nucleic acid metabolism, protein synthesis, and bone formation in vitro and in vivo [377]. Incorporating Zn into hydroxyapatite (HA) has been found to improve the bioactive properties of these materials [378].

The Ca/P ratio influences the incorporation of Zn into HA films. Apatite readily dissolves into β-tricalcium phosphate (β-TCP) and octacalcium phosphate, while the addition of Zn enhances the material’s mechanical properties [379,380].

We investigated the electrochemical behavior of Ti-6Al-4V subjected to PEO in solutions containing Ca, P, and Zn ions. Increasing the concentration of Zn ions led to an increase in pore pores on the PEO films, although the pore size slightly decreased. XRD analysis of the PEO-treated films showed a strong anatase phase peak alongside a weaker rutile phase peak.

**Figure 20:**
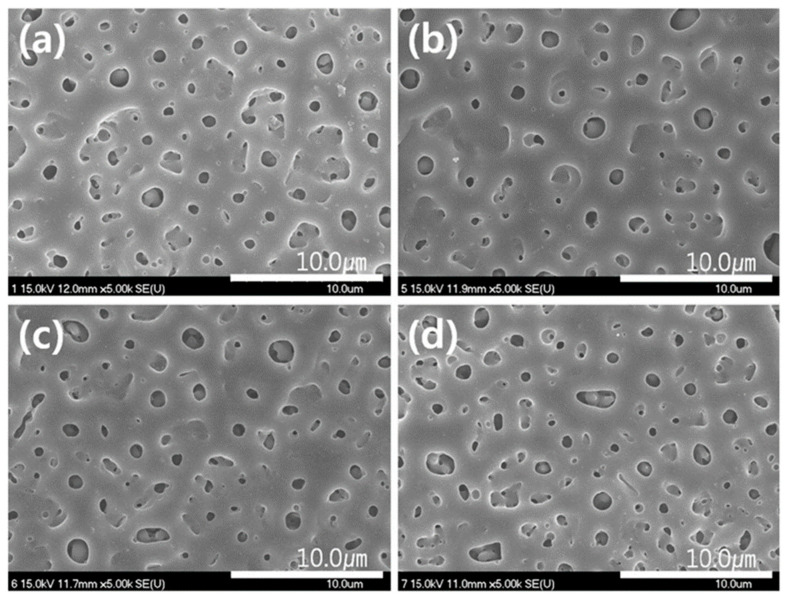
FE-SEM images show PEO-treated film surfaces of (**a**) Z0, (**b**) Z5, (**c**) Z10, and (**d**) Z20 specimens. Reproduced with permission [376].

Figure 20 presents FE-SEM images of PEO films formed on Ti-6Al-4V at 280 V using different electrolytes. Images (a), (b), (c), and (d) correspond to coatings Z0, Z5, Z10, and Z20, respectively, showcasing uniformly porous surfaces [376].

The porous structures exhibited significant roughness due to the formation of microdischarge channels during the PEO process. Numerous sparks were observed on the coating surface when the applied voltage surpassed the critical threshold necessary to penetrate the barrier layer. This process resulted in a surface characterized by crater-like holes formed from the ejection and deposition of molten materials [381].

None of the PEO films showed evidence of microcracks. The number of pores within a 10 μm × 10 μm 10 μm × 10 μm area ((10 μm) 2 (10 μm) 2) was quantified using a specific surface area measurement through an analog analyzer (Image J, Wayne Rasband, Bethesda, MDA, USA, https://imagej.net/ij/ accessed on 19 December 2024). The findings revealed that pore growth diminished as the concentration of Zn ions in the electrolyte increased. However, it is noteworthy that high zinc concentrations can be toxic to human tissues [382]. Consequently, it is hypothesized that bone apatite does not form in the case of Z20 in simulated body fluid (SBF) due to the cytotoxic effects associated with elevated Zn content.

**Figure 21:**
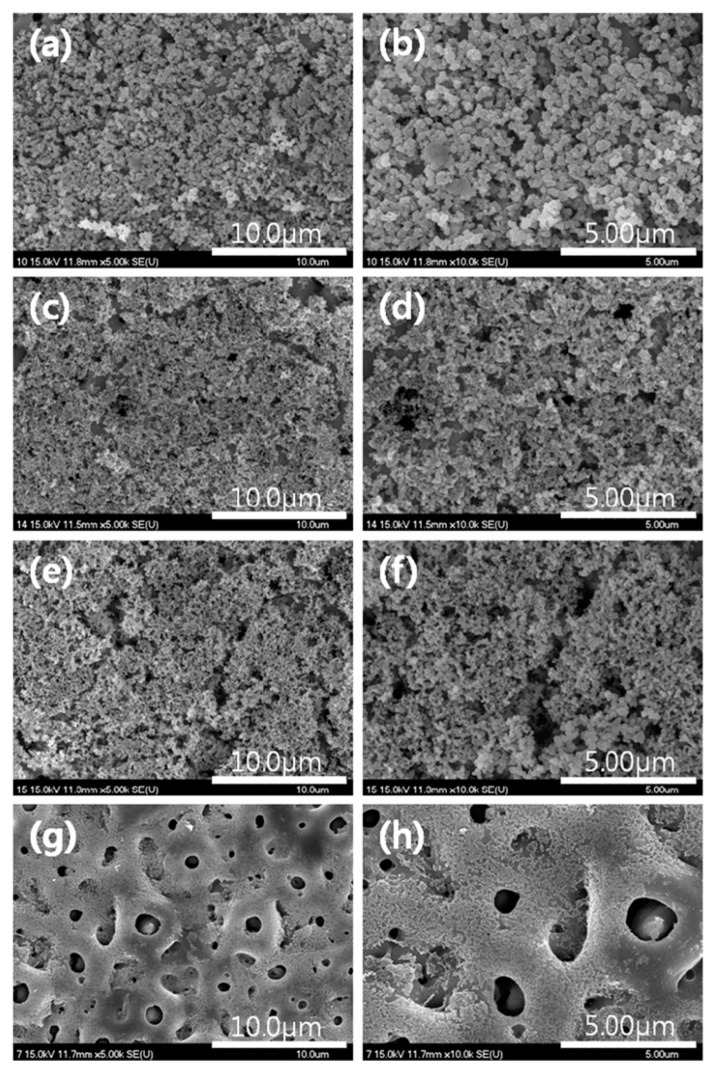
FE-SEM images showing the morphology of bone-like apatite: (**a**) Z0, (**b**) Z0 (magnified—a), (**c**) Z5, (**d**) Z5 (magnified—**c**), (**e**) Z10, (**f**) Z10 (magnified—**e**), and (**g**) Z20, (**h**) Z20 (magnified—**g**). Reproduced with permission [376].

Figure 21 displays FE-SEM images of PEO films containing Zn, formed on Ti-6Al-4V surfaces in SBF solution over 12 h. Images (a) through (d) show surfaces at ×5000 magnification for Z0, Z5, Z10, and Z20, respectively, while images (e) through (h) represent the same samples at ×10,000 magnification. These images indicate that bone apatite forms effectively in SBF for all samples except for the Z20 coating due to its high Zn content [376].

The Z20 samples formed less bone apatite than other samples, likely due to the cytotoxic effects of high Zn concentrations, which can negatively impact bone apatite formation when the Zn content reaches 20% [380,383]. However, successful bone apatite formation was observed in the Z10 samples immersed in SBF.

Bioactive materials can be incorporated into the surface layer during the PEO process by introducing them into the electrolyte solution [384]. This makes surface deposition methods based on PEO particularly promising for forming HA coatings, including hybrid coatings with TiO_2_ [385].

To evaluate the corrosion resistance and electrochemical properties of TiO_2_:n-HA coatings compared to uncoated titanium, the PEO method was applied to Ti-6Al-4V alloy substrates. The HA nanopowder used, nanoXIM-HAp303, was sourced from Fluidinova (Moreira da Maya, Portugal) as a water-based paste with a solid content of 30%. The nanopowder met the requirements for hydroxyapatite specified for surgical implants (ISO13779 and ASTM F1185) [385]. The substrates were treated with PEO for 10 min in an electrolyte solution containing 6 g/L disodium hydrogen phosphate (Na_2_HPO₄, Fisher Scientific, Hampton, NH, USA) and 10 g/L nanoXIM-HAp303 at room temperature.

A two-step current regime was employed. First, potentiostatic polarization at U(+) = 250 V was applied for 15 s to form a uniform primary oxide film. This was followed by galvanostatic polarization using pulsed bipolar current mode with current densities of i(+) = 3 5 mA/cm^2^ and i(−) = 17.5 mA/cm^2^.

Electrochemical testing was performed in buffered HBSS (Sigma-Aldrich, St. Loui, MO, USA, H1387) at room temperature using a Biologic SP-150 system with a three-electrode cell: the working electrode was a 6 cm^2^ sample, the counter electrode was a graphite rod, and the reference electrode was Ag/AgCl with 3.5 M KCl. Results confirmed the stability of the TiO_2_ + HA surface, which exhibited a two-layer structure. The inner barrier layer protected corrosion, while the outer porous layer enhanced cell adhesion.

**Figure 22:**
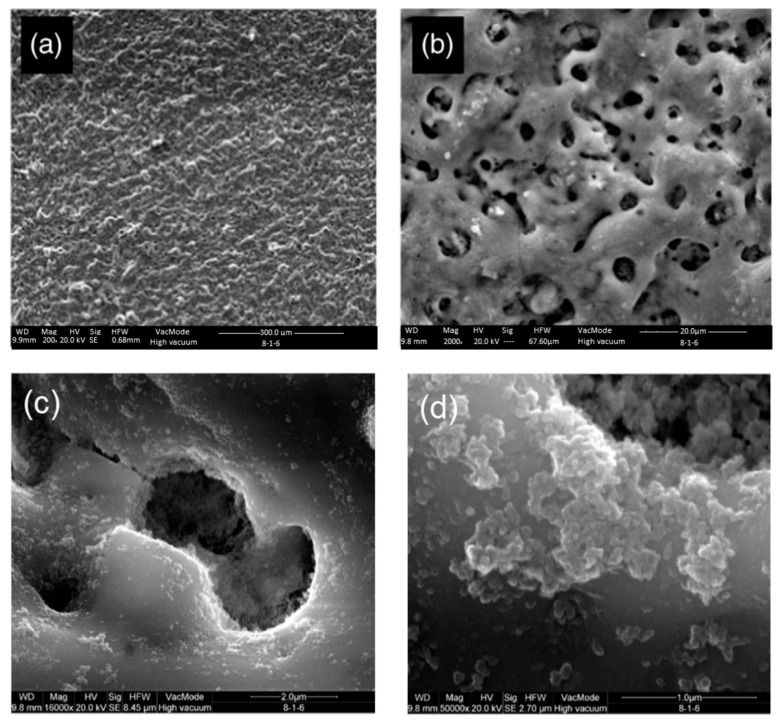
SEM micrographs of the samples after the PEO treatment: general view of the surface (**a**), porous structure of the surface (**b**), interconnecting pores (**c**), and HA nanoparticle agglomerates on the porous surface (**d**). Reproduced with permission [385].

SEM micrographs of the PEO-treated specimens revealed a porous surface morphology, with pores averaging 0.5–4 μm, likely corresponding to the TiO_2_ phase (Figure 22) [385]. Although the plasma spraying technique for applying hydroxyapatite coatings has been extensively studied, it is known to result in coatings with low adhesion strength and significant biodegradation [386].

Substrates used were pure titanium (>99 at.%) with a diameter of 15 mm. The surfaces were polished with abrasive paper, rinsed with distilled water, ultrasonically cleaned with acetone and deionized water, and dried in a desiccator. A DC switching power supply was used for the PEO process, with a titanium disk as the anode and a stainless-steel plate as the cathode. The electrolyte was an aqueous solution containing calcium and phosphate salts.

The applied voltage ranged between 240 V and 450 V, producing layers approximately 10 μm thick. SEM and XRD analyses were conducted to assess surface morphology and composition. Tensile strength was evaluated using an Instron 1195 test system, and nanoindentation measurements were performed to determine the elastic modulus. Residual stress was analyzed using two-dimensional X-ray diffraction.

Samples prepared at 240 V and 350 V exhibited stronger adhesion between the coating and substrate than those prepared at higher voltages. Elastic modulus and residual stress were found to increase with applied stress. The elastic modulus of the porous layer was significantly lower than that of pure titanium, indicating distinct mechanical properties.

- The morphological characteristics of the surface during PEO are significantly influenced by the spark’s size and shape, as well as the chemical composition of the anodizing solution.
- Anodic coatings produced in the P-Si solution exhibited lower porosity than those formed in other solutions, enhanced corrosion resistance, and increased hardness. Conversely, coatings created in the P-S solution demonstrated high surface porosity, with a morphology resembling bone structures. In the P solution, circular pore structures were predominantly observed.
- The anatase crystalline phase was the dominant structure in anodic coatings developed with the P and P-Si electrodes, with only a tiny amount of the rutile phase present. In contrast, the primary crystalline phase in coatings formed in the P-S solution was distinctly different.
- Potentiostatic coatings outperformed those produced under galvanostatic control in terms of tribological properties. This was particularly evident in the anodic coatings obtained in the P solution at 250 V and in the P-Si solution at 400 V, both of which exhibited the lowest wear rates [387].

## 7. PEO Using Microparticles and Elements

PEO is a high-voltage anodizing technique performed at potentials exceeding the dielectric breakdown of the coating. This process generates short-lived microdischarges on the material’s surface, accompanied by gas evolution [388]. The localized heating and compressive stresses during PEO promote crystallization within the anodic coating. Simultaneously, ionic incorporation from the electrolyte modifies the chemical composition and crystalline structure of the oxide layer. As the coating develops and thickens, the surface topography evolves, enabling the creation of coatings with specific functionalities [389]. The process parameters, including the anodizing solution’s composition, concentration, pH, temperature, processing time, and electrical settings (voltage and current density), play a crucial role in determining surface characteristics [390].

Alkaline solutions are frequently employed in PEO to enhance the tribological properties of titanium alloys by forming thick coatings [391]. However, the use of sodium hypophosphite as a phosphorus source in anodic coatings has been relatively underexplored. In one study, anodic films were prepared on Ti_6_Al4V substrates in sodium-hypophosphite-based electrolytes under galvanostatic and potentiostatic modes with varying electrical parameters. EIS analysis was used to establish correlations between the electrical parameters and plasma characteristics. The substrates, measuring 10 × 10 × 1 mm, were mechanically polished (average roughness Sa = 188.84 nm), cleaned ultrasonically in acetone for 900 s, and chemically purified in an alkaline solution of H_2_O_2_ (25 g/L) and NaOH (32 g/L) at 60 °C for 900 s before rinsing with distilled water and drying in cold air.

The anodizing process was performed using a KPCK BHK 500-0.4 MG power supply (Kepco, Inc., New York, NY, USA) in a 100 mL electrochemical cell cooled in a water bath, with a stainless-steel cathode. Calcium was included in the solution to enhance the wear resistance of biomedical coatings. In P-Si solutions, silicate additions improved wear resistance [392,393], while sulfate ions in P-S solutions promoted the formation of rutile phases, enhancing wear resistance and acting as a solid lubricant [394,395]. Galvanostatic coatings were produced using direct current with time-varying potential.

Bakin et al. [396] highlighted that HAp ceramics can be alloyed with ions naturally present in bones and teeth to improve bioactivity, mechanical strength, and osseointegration. Magnesium, an essential element constituting 1–1.5% of bone tissue, contributes to strength, supports HA crystal growth, and enhances cell vitality [397]. Silicon plays a crucial role in bone tissue development by aiding collagen synthesis during early bone formation and initiating organic matrix mineralization. Si-containing calcium phosphate ceramics have demonstrated superior biological activity and enhanced osteoblast attachment and proliferation on surfaces [398].

One of the challenges in surgical implantation is the risk of infection, which can jeopardize implant success [399]. Silver coatings provide antiseptic properties, reducing the risk of inflammation and improving the integration of the implant with surrounding bone tissue. The incorporation of silver into coatings has been widely reported [400]. However, Kim et al. [401] noted limitations in using metallic silver or silver salts as antimicrobial agents due to solubility issues that hinder long-term release of silver ions. Efforts to improve the antibacterial activity of PEO-treated titanium and its alloys include incorporating Ag nanoparticles into coatings [402,403,404].

The microplasma process during PEO involves oxidation of the metal surface, decomposition of the electrolyte, and synthesis and deposition of compounds from the electrolyte onto the substrate. Calcium phosphate microplasma coatings are typically derived from electrolytes containing soluble or insoluble calcium and phosphorus compounds. Homogeneous electrolytes improve the uniformity of coating composition, thickness, and morphology while mitigating issues associated with heterogeneous electrolytes, such as particle distribution and interaction imbalances. Alkaline homogeneous electrolytes with pH < 9 enable coatings with Ca/P ratios between 1.1 and 4.0 [398], although most applications require coatings with a Ca/P ratio close to the stoichiometric value of 1.67 for HA. For pH > 10, coatings with Ca/P ratios near 1.5 are achievable, closely matching the characteristics of natural bone [404,405].

The inclusion of additives such as magnesium (Mg), silicon (Si), and silver (Ag) in homogeneous electrolytes enhances coating performance. Magnesium improves coatings’ mechanical properties and bioactivity [397], while silicon facilitates new bone tissue growth [406]. Silver provides antimicrobial properties critical for reducing infection risks [399].

Heterogeneous electrolytes, comprising phosphoric acid solutions and insoluble powders like hydroxyapatite or calcium carbonate, allow for customized coating characteristics but present challenges, including poor deposition control and particle interaction inconsistencies. Homogeneous electrolytes avoid these drawbacks, providing consistent results over extended operations [407].

Magnesium-containing coatings primarily consist of MgCO_3_, while coatings without Mg^2^⁺ generally include Ca_3_(PO_4_)_2_ and HA. Si and Ag additions produce coatings with phases like rutile, anatase, and titanite (CaTiSiO_5_). Elemental analyses confirm the incorporation of Si and Ag into coatings formed from silicon- and silver-containing electrolytes [408].

## 8. PEO—Aspects of Surface Strength

Calcium-phosphate-based composites, including hydroxyapatite (HA) and apatite carbonate (CA), have recently gained attention as biocompatible and desirable coating materials for clinical and biomedical applications. A critical factor in developing PEO surfaces is the influence of voltage, processing time, and electrolyte composition on forming calcium phosphate composite layers on biomedical substrates.

These parameters significantly affect the coatings’ structure, morphology, pH, thickness, and crystallinity, tailoring them for various technical and biomedical applications. The resulting layers, with 10 to 20 µm thicknesses, were evaluated for their physical, chemical, mechanical, and tribological properties. This evaluation aimed to understand how the applied parameters and electrolyte compositions impact the coatings’ surface morphology and phase composition.

It was observed that during PEO, the concentrations of calcium, phosphorus, and titanium in the coating increased, enhancing and strengthening the oxide layer’s thickness. Studies have also shown that heat treatment can alter the composite layer’s crystallinity, thickness, and composition. The corrosion resistance of these coatings was investigated using potentiodynamic polarization tests in an environmental model. Optimal corrosion resistance was achieved for coatings processed at 500 V for 15 min in Ringer’s solution.

This research paves the way for advancing PEO technology to produce more sophisticated titanium-based implants with enhanced mechanical and biomedical properties [409]. Titanium and its alloys are widely utilized in biomedical fields due to their minimal adverse tissue reactions, making them suitable for applications such as artificial joints, bone endoprostheses, and implants [410]. The naturally occurring oxide layer on titanium surfaces promotes favorable biological responses [4].

While Ti-6Al-4V is commonly used for biomedical designs, concerns about releasing toxins and ions—particularly vanadium—pose long-term risks. Modification of the surface composition has been implemented to mitigate these adverse effects, thereby improving biocompatibility. These modifications also enhance mechanical properties such as strength, Young’s modulus (~100 GPa), yield strength (~650 MPa), and corrosion resistance [411].

**Figure 23:**
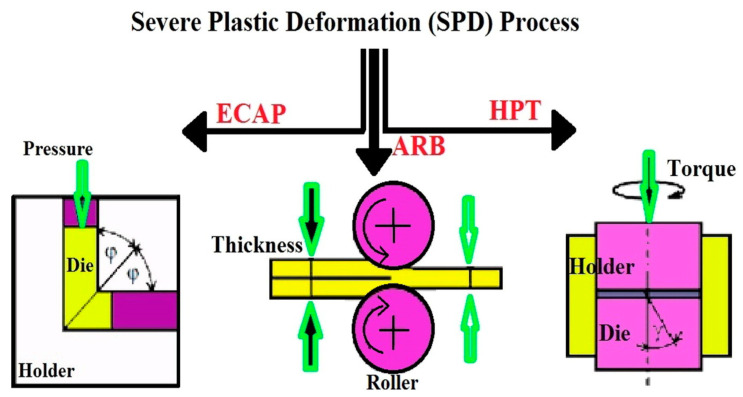
Schematic view of the SPD process. Reproduced with permission [414].

To produce high-strength and homogeneous titanium-based substrates, severe plastic deformation (SPD) techniques, including angular pressing [412], hot and cold forging, and equal channel angular extrusion [413], are employed. Figure 23 illustrates a schematic representation of these processes [414].

The next step in improving titanium-based implants focuses on developing bioactive coatings to enhance their biological performance. These coatings are designed to elicit specific biological responses from the implant, aligning with human bone tissue’s mechanical and chemical properties [414].

**Figure 24:**
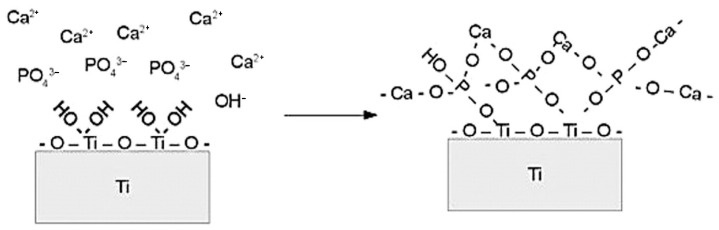
Schematic view of bonding hydroxyapatite on the titanium surface. Reproduced with permission [415].

Hydroxyapatite (HA), with the chemical formula Ca_10_(PO_4_)_6_(OH)_2_, is a calcium phosphate compound that constitutes over 65% of the weight of human bone tissue. HA can be deposited onto titanium surfaces through a mechanistic reaction between an aqueous solution of calcium hydroxide and phosphoric acid under ambient conditions. The reaction proceeds according to the chemical equation depicted in Figure 24 [272,415].

**Figure 25:**
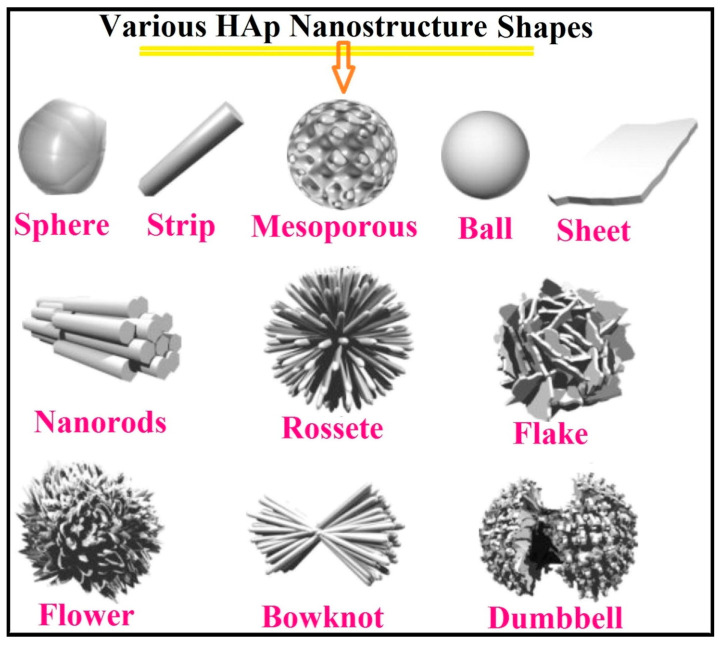
Schematic view of various HAp nanostructures. Reproduced with permission [417].

Hydroxyapatite (HA) exhibits a hexagonal symmetrical structure that can vary between needle-like and lamellar formations depending on the application conditions and adjustments to its stoichiometric ratio (Ca:P = 1.67). Studies have demonstrated HA’s ability to promote new bone formation through osteoconduction without causing local or systemic toxicity or other adverse effects on the body [414,416,417] (Figure 25).

ND nanoparticles with grain sizes smaller than 100 nm exhibit superior stoichiometry, morphology, and purity compared to other ND structures [418].

**Figure 26:**
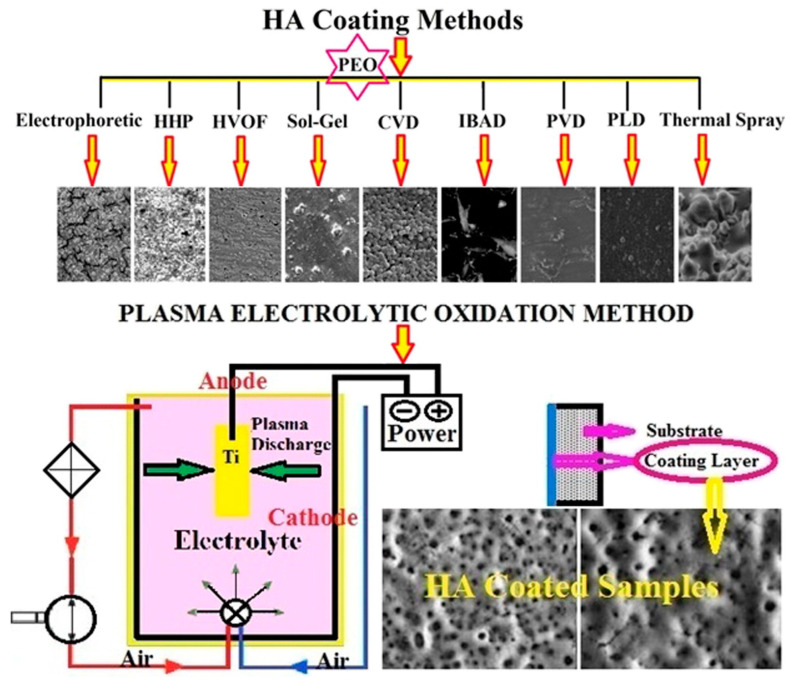
Morphology of the HA layer via different coating methods and schematic view of the PEO method. Reproduced with permission [419].

Moreover, HA and other calcium-phosphate-based composite layers can be applied to titanium surfaces using a variety of techniques, including electrophoretic deposition (EPD), hydrothermal hot pressing (HHP), high-velocity oxygen oxidation (HVOF), sol-gel processes, chemical vapor deposition (CVD), ion-beam-assisted deposition (IBAD), physical vapor deposition (PVD), pulsed laser deposition (PLD), thermal spray methods, and plasma electrolytic oxidation (PEO), which encompasses microarc oxidation (MAO). Figure 26 illustrates the diverse HA nanostructures synthesized through these methods [414,419].

## 9. Plasma Electrolytic Oxidation Method Calcium-Phosphate-Base Composite Layer via PEO

Compared to other coating techniques, PEO offers a unique advantage by enabling the incorporation of ions such as calcium, phosphorus, and titanium dioxide into a composite layer. Modifying the surface’s crystallinity and morphology enhances its biocompatibility and mechanical properties [420,421]. Additionally, PEO can create coatings on substrates with complex geometries, improving adhesive strength.

The high temperatures generated by plasma discharges during PEO facilitate the transformation of particles into crystalline phases, which exhibit superior strength and hardness. Furthermore, PEO enables the formation of coating layers with adequate electrical conductivity. The method also allows for integrating calcium and phosphorus ions into titanium and its alloys by fine-tuning coating parameters such as electrolyte composition, voltage, current density, and processing time. Table 3 summarizes the outcomes of various PEO approaches used to produce bioceramic layers, such as HA and CA, on titanium surfaces [414].

The electrolyte used in the PEO process must ensure a uniform distribution of crystals on titanium surfaces, potentially enhancing the adhesion between the HA coating layer and pure titanium. Most research suggests suspending titanium in electrolytes containing calcium acetate and glycerophosphate or β-glycerophosphate to produce bioceramic films like HA [366]. In some studies, calcium acetate has been substituted with calcium chloride [313] or trisodium phosphate [424]. Additionally, β-glycerophosphate has been replaced in certain cases with β-calcium glycerophosphate [312], monobasic potassium phosphate, or monobasic sodium phosphate dihydrate [385].

To achieve ND coatings with appropriate stoichiometry, a neutral or acidic electrolyte with a sufficiently high pH is recommended for the PEO method [425]. Previous studies have shown that HA remains stable in aqueous electrolytes with pH values of 4.2 and 6.0. Unlike traditional anodizing, which is typically performed at low voltages (5–50 V) under static or stirred conditions, PEO operates at significantly higher voltages (100–600 V) using alternating current [272].

## 10. PEO—Antibacterial Effect

Among the various strategies for enhancing antibacterial activity, modifying the surface of titanium-based medical implants by incorporating antimicrobial agents into the surface layer is an effective, cost-efficient, and straightforward approach [426]. One common method involves the supplementation of the surface with bactericidal agents.

Antibacterial agents suitable for metal medical implants include antibiotics [11] or inorganic bactericides such as silver, carbon, zinc, copper, and compounds like titanium dioxide, zinc oxide, tantalum nitride, titanium nitride, and zirconium nitride [427]. A critical requirement for any antibacterial surface treatment is that the added components should not interfere with the integration of the implant with surrounding tissue.

While the positive impact of hydroxyapatite (HA) on osseointegration is well established, its antibacterial properties remain a topic of debate. Some studies (e.g., [427,428]) suggest that HA exhibits antibacterial activity, whereas others indicate that HA may facilitate bacterial biofilm formation near the implant surface [429].

Modifying the surface of medical implants to enhance antibacterial properties is a recognized approach [9]. This can involve incorporating antibiotics [135] or inorganic antibacterial compounds, such as silver, carbon, zinc, copper, and various oxide and nitride-based materials (e.g., titanium oxide, zinc oxide, tantalum nitride, titanium nitride, and zirconium nitride). However, it is essential that such modifications do not compromise the integration of the implant with surrounding tissue.

Although the role of HA in improving osseointegration is well documented, its influence on bacterial activity is less clear. Some researchers have reported that HA has inherent antibacterial properties [67], while others have suggested that it may promote the development of bacterial biofilms near the implant surface [135].

## 11. PEO with the Inclusion of Nanocomponents

Nanoscience focuses on the study of objects with dimensions ranging from a few to several hundred nanometers [430]. Examples of materials within this scale include colloids, micelles, polymer molecules, buckytubes, buckyballs, quantum dots, phase-separated polymers, self-assembled monolayers, block copolymer domains, large molecules, or aggregates of molecules [431]. At the nanoscale, surface and interfacial properties become critical, as nearly 90% of biological reactions occur at the substrate surface [432]. Nanostructured materials exhibit significantly increased surface areas compared to macroscopic materials, with a substantial proportion of their atoms located at the surface. This results in structures where nearly every atom is interfacial, directly influencing the material’s macroscopic properties [433].

Nanocoatings not only fulfill their primary roles, such as etch protection and corrosion resistance, but also offer secondary functionalities, including drug delivery and enhanced biocompatibility. Self-assembled monolayers (SAMs) serve as a notable example of nanostructures, enabling biomaterial surface modifications for specific clinical applications. Another example is carbon nanotubes (CNTs), which, due to their unique structural, electrical, and mechanical properties combined with their low weight and size, have become transformative materials in nanotechnology and materials science. In recent years, CNTs have been extensively investigated for biological and medical applications because of their high reactivity in facilitating cell attachment and protein synthesis [434]. Titanium, its alloys, and related materials can benefit significantly from the development of nanostructured surfaces, improving their biological properties and expanding their clinical applications. Enhanced osseointegration of biomaterials is crucial for reducing implant rejection rates and improving patients’ quality of life [434].

The ability of nanostructured materials to modulate cellular responses has led to an increase in research and publications in this field [435]. Incorporating nanotopographic features that mimic the natural bone structure has become a promising direction in tissue engineering [436]. Studies suggest that nanostructured biomaterials may exhibit surface and chemical properties similar to native bone, making them ideal substrates for bone regeneration [437]. Nanopatterns come in various shapes (e.g., cylinders, pyramids) and dimensions (height, width, depth, and spacing), depending on the fabrication technique. Not only does surface nanotopography influence stem cell fate, but high-aspect-ratio nanoparticles have also been shown to possess antibacterial properties [438], including the prevention of biofilm formation [439].

Antibacterial activity on biomaterial surfaces is especially critical during the initial hours after implantation, referred to as the “race for the surface” [152]. If germicidal surfaces inhibit bacterial adhesion during this time, host cells are more likely to colonize the surface, reducing the need for prolonged antibacterial protection. Studies show that mammalian cells can dominate the surface in the long term. For instance, Pham et al. demonstrated that eukaryotic cells could proliferate on pre-infected nanobar surfaces immediately after bacterial growth was inhibited [153]. Mechanosensory pathways in eukaryotic and prokaryotic cells differ [151], paving the way for the design of nano-coated surfaces that selectively support eukaryotic cell attachment and proliferation while preventing bacterial colonization. Nanoparticles that initially kill bacteria and subsequently promote host cell attachment and growth could offer significant long-term advantages.

Understanding the antibacterial behavior of nanomaterials requires distinguishing surface chemistry effects from those of nanostructuring. For example, naturally occurring germicidal surfaces often exhibit hydrophobicity and low surface energy, properties that vary with nanoparticle size [440]. Coated surfaces have shown antibacterial behavior, suggesting that bactericidal effects may arise from physical properties. Hydrophilic surfaces have also demonstrated antibacterial activity [441]. When nanomaterials are made from inherently antibacterial materials, such as TiO_2_ or ZnO, it can be challenging to separate the effects of nanostructuring from the material’s intrinsic properties. In many cases, these effects work synergistically to enhance bactericidal behavior, which often increases with bacterial adherence to the surface [442]. The mechanical disruption of bacteria by nanoparticles can vary depending on the bacterial maturity stage [443]. Importantly, nanomaterials tend to influence adhesion forces more than the quantity of adhering bacteria [444].

The advent of femtosecond lasers marked a new era in micro- and nanomachining. Laser-induced periodic surface structures (LIPSSs), or ripples, are periodic features formed on material surfaces after exposure to laser pulses near the ablation threshold [156]. Since their discovery approximately five decades ago by Birnbaum [157], LIPSSs have become a significant research topic, with ultrafast laser pulses, especially femtosecond pulses, demonstrating versatility in generating these structures. LIPSS can be applied to various materials, including metals [445], semiconductors [161], dielectrics [446], ceramics [159], and polymers [158], when irradiated near their ablation threshold.

LIPSSs offer extensive applications in biomedical surface topography [162], incandescent surface light sources, photoelectronic emissions [447], surface wettability modification [448], metal and silicon blackening [449], and the creation of nanostructures for surface coloring [450].

## 12. Conclusions and Future Perspectives

This review underscores the potential of plasma electrolytic oxidation (PEO) in creating bioceramic coatings, such as hydroxyapatite (HA) and calcium phosphate, for titanium-based implants, offering enhanced biocompatibility and antibacterial properties. Incorporating bioactive elements like calcium, phosphorus, and zinc into the coating matrix has improved osseointegration and surface durability, demonstrating the synergy between material composition and functional performance. Advances in nanotechnology, including nanotubes and nanostructures, have further highlighted their ability to mimic natural bone properties, support cell proliferation, and reduce bacterial colonization.

Future research should optimize electrolyte formulations and PEO processing parameters to achieve improved coating uniformity, reduced porosity, and enhanced mechanical and antibacterial properties. Long-term clinical studies are necessary to evaluate these multifunctional coatings’ durability, biocompatibility, and biofilm resistance. Emerging nanofabrication techniques, such as laser-induced periodic surface structures (LIPSS) and multifunctional nanoparticles, are promising for designing next-generation implant surfaces tailored to specific biomedical applications.

## Figures and Tables

**Table 1:** Particles that have been introduced into the PEO electrolyte.

Particles	Properties and Field of Applications	Reference
Polytetrafluoroethylene	Lower friction coefficient, chemical inertness and hydrophobicity	[224]
Ag	Antibacterial activity	[225]
Hydroxyapatite (HA)	Osteogenesis and biomaterial	[226]
MoS_2_	Solid lubricant	[227]
Clay minerals	Absorption capacities and filler material	[217]
ZrO_2_ (monoclinic, tetragonal, and cubic)	High chemical stability	[228,229]
SiO_2_	High heat and chemical resistance	[230,231]
TiO_2_	High chemical stability and heat resistance	[232]
Si_3_N_4_	High hardness and wear resistance	[233]
Al_2_O_3_	High hardness and insulator	[232]
CeO_2_/Ce_2_O_3_	High chemical stability, superconductors and sensors	[234,235]
SiC	High mechanical strength and chemical inertness	[236,237]
Graphite	Solid lubricant	[238]
Calcium phosphates	Natural bone component	[239]
Fe/Fe_2_O_3_	Ferromagnetic material	[240]
Co	Ferromagnetic material	[241]
Cu	Antibacterial activity	[242]
Ni/NiO, MnO_2_/Mn_2_O_3_	Catalytic activity	[243]

**Table 2:** Material surfaces coated with proteins.

Surface	Protein	Study	Investigations	Results	Literature
TiUnite	rhBMP-2	In vivo	TiUnite-coated screw implants in 12 Labrador dogs	TiUnite surfaces coated with rhBMP-2 possess significant potential to stimulate bone growth	[333]
TiO_2_	BMP-2	In vitro	Human osteoblasts growth on surfaces: (non)anodized (un)coated Ti plates	Anodized surfaces coated with BMP-2 induced better osteoblast adhesion	[334]
Ti cp and Ticer	BSP, Collagen type I Fibronectin	In vitro	Materials’ influence on adult human maxillary bone cells’ behavior	Coating Ti cp induces better biological properties than a rough ceramic surface material; the best improvement for materials coated with BSP	[335]
Ti cp and Ticer	BSP, Collagen type I	In vitro	Effect of protein coated surfaces on bone-derived cells	Collagen surfaces—unsuitable for the cell attachment; BSP surfaces—advance osteoinduction process	[336]

**Table 3:** Parameters used for producing the calcium-phosphate-base composite on titanium.

Ti Alloy	Electrolyte	Voltage (V)	Time (min)	XRD Detected Phase	Preheat, Oxidation Annealing Temp (°C)	Literature
Cp_2_Ti	Ca(CH_3_COO)_2_, 0.028–0.085 M Na β-glycerophosphate, 0.005–0.02 M	350	3	Ti TiO_2_ Anatase HAα-TCP CaTiO_3_	No Preheating Oxidation at 70 ± 3 No heat treatment	[366]
Ti_6_Al_4_V	Ca(CH_3_COO)_2_·H_2_O, 0.26 M Na_2_HPO_4_·2H_2_O, 0.12 M	400	15	TiO_2_ Anatase TiO_2_ Rutile TiV Al_0.3_Ti_1.7_ HA	No preheating Oxidation at room temperature No heat treatment	[422]
Ti_6_Al_4_V	Ca(CH_3_COO)_2_·H_2_O, 0.26 M Na_2_HPO_4_·2H_2_O, 0.12 M	400	60	TiO_2_ Anatase TiO_2_ Rutile TiV Al_0.3_Ti_1.7_ HA CaTiO_3_ Al_2_O_3_ Ca_10_(PO_4_)_6_(OH)_2_	No preheating Oxidation at room temperature No Heat treatment	[422]
Cp_2_Ti	Ca(CH_3_COO)_2_, 0.015 mol/L Ca β-glycerophosphate, 0.02 mol/L	450	7.5	Ti TiO_2_ Anatase TiO_2_ Rutile HA	Preheated at 300 Oxidation at room temperature Heat treatment for 10 h at 190 with autoclave	[423]
Cp_2_Ti	Ca(CH_3_COO)_2_, 0.03 M Ca β-glycerophosphate, 0.02 M	400	60	TiO_2_ Anatase TiO_2_ Rutile Ca_2_Ti_2_O_6_	No preheating Oxidation at 15 Heat treatment for 4 h at 220 with autoclave	[423]
Cp_2_Ti	Ca(CH_3_COO)_2_, 0.2 mol/L Ca β-glycerophosphate, 0.02 mol/L	350	3	TiO_2_ Anatase TiO_2_ Rutile HA	No preheating Oxidation at 70 ± 3 No heat treatment	[272]
Cp_2_Ti	Ca(CH_3_COO)_2_, 0.2 mol/L Ca β-glycerophosphate, 0.02 mol/L	350	6	TiO_2_ Anatase TiO_2_ Rutile HA CaTiO_3_ α-TCP	No preheating. Oxidation at 70 ± 3. No heat treatment.	[272]
Cp_2_Ti	Ca(CH_3_COO)_2_, 0.2 mol/L Ca β-glycerophosphate, 0.02 mol/L	350	10	TiO_2_ Anatase TiO_2_ Rutile HA CaTiO_3_	No preheating Oxidation at 70 ± 3 No heat treatment	[272]

## Data Availability

No new data were created or analyzed in this study. Data sharing is not applicable to this article.
